# Liver Transplantation and Hepatitis C

**DOI:** 10.1155/2012/686135

**Published:** 2012-07-26

**Authors:** Nobuhisa Akamatsu, Yasuhiko Sugawara

**Affiliations:** ^1^Department of Hepato-Biliary-Pancreatic Surgery, Saitama Medical Center, Saitama Medical University, 1981 Tsujido-cho, Kamoda, Kawagoe, Saitama 350-8550, Japan; ^2^Artificial Organ and Transplantation Division, Department of Surgery, Graduate School of Medicine, The University of Tokyo, 7-3-1 Hongo, Bunkyo-ku, Tokyo 113-8655, Japan

## Abstract

Hepatitis-C-virus- (HCV-) related end-stage cirrhosis is the primary indication for liver transplantation in many countries. Unfortunately, however, HCV is not eliminated by transplantation and graft reinfection is universal, resulting in fibrosis, cirrhosis, and finally graft decompensation. The use of poor quality organs, particularly from older donors, has a highly negative impact on the severity of recurrence and patient/graft survival. Although immunosuppressive regimens have a considerable impact on the outcome, the optimal regimen after liver transplantation for HCV-infected patients remains unclear. Disease progression monitoring with protocol biopsy and new noninvasive methods is essential for predicting patient/graft outcome and starting antiviral treatment with the appropriate timing. Antiviral treatment with pegylated interferon and ribavirin is currently considered the most promising regimen with a sustained viral response rate of around 30% to 35%, although the survival benefit of this regimen remains to be investigated. Living-donor liver transplantation is now widely accepted as an established treatment for HCV cirrhosis and the results are equivalent to those of deceased donor liver transplantation.

## 1. Introduction

End-stage liver disease caused by chronic hepatitis C virus (HCV) infection is the leading cause of liver transplantation in developed countries [1, 2], including Japan [3]. Unfortunately, liver transplantation does not cure HCV-infected recipients, but reinfection of HCV universally occurs and disease progression is accelerated compared with that in the nontransplant population, resulting in poor outcomes for HCV-infected recipients. Although several studies have investigated the factors affecting the natural history of recurrent HCV, many aspects remain unclear and require further investigation [4]. For patients with progressive fibrosis, it is essential to monitor disease progression and the only strategy that is known to modify the outcome is antiviral therapy at an appropriate disease stage. In this paper, we address the issues that transplant physicians face in the management of patients with recurrent hepatitis C, review the results of antiviral treatments, and discuss on living donor liver transplantation (LDLT) for HCV cirrhosis.

## 2. Natural History of Hepatitis C after Liver Transplantation

HCV reinfection of liver allografts is universal, occurring just after reperfusion followed by a rapid increase in HCV ribonucleic acid (RNA) levels within 4 postoperative months [5]. Diagnosis of recurrent HCV infection is based on the detection of HCV RNA in the serum and/or liver graft, but diagnosis of recurrent disease requires histologic confirmation [6]. The histologic features of liver injury usually resemble those of nontransplant HCV hepatitis typically developing after 3 months, but the clinical presentation, severity, and outcome are extremely heterogeneous and more profound compared to those in immune competent patients [7]. The pattern of recurrence is worse over time compared with chronic hepatitis, and further cirrhosis, as well-described in the nontransplant population, develops with higher viremia and faster fibrosis progression. Progression to cirrhosis usually takes 9 to 12 years after liver transplantation with a linear progression of histologic fibrosis [7, 8]. A less common, but well-documented form of recurrence is called fibrosing cholestatic hepatitis (<10%), possibly mediated by a direct cytopathic mechanism under an extremely high viral load and immune-compromised condition. Graft failure occurs in 50% of recipients within a few months after fibrosing cholestatic hepatitis develops [9]. Some HCV-reinfected recipients, however, show no apparent disease progression for at least the first decade and their graft injury remains mild or even absent despite a high viral burden.

Overall, cirrhosis develops in approximately 25% of liver transplant recipients (range 8%–44%) after 5 to 10 years and this percentage is likely to increase with an increase in the follow-up period [7, 8]. Once cirrhosis is complete, survival time is severely decreased and decompensation is encountered with cumulative rates at 1 and 3 years of 40% and 60%, respectively, which finally results in graft failure [8, 10].

The development of decompensated cirrhosis due to recurrent hepatitis C is now the most frequent cause of graft failure, patient death, and the need for retransplantation in HCV-infected recipients [6, 8, 10–13]. As a result, survival is significantly decreased compared with other indications, an overall 10% difference at 3 years. In the most recent United Network for Organ Sharing/Organ Procurement and Transplantation Network (UNOS/OPTN) study from the United States, 3-year survival is 78% among 7459 HCV-positive recipients compared with 82% among 20734 HCV-negative recipients (*P* < 0.0001; http://www.unos.org/) [14].

The poor outcome of HCV-positive recipients has resulted in the divergence in transplant outcomes between HCV-positive recipients and HCV-negative recipients. Improvements in organ preservation, surgical techniques, and postoperative care have dramatically improved the survival of HCV-negative recipients over the last two decades, whereas this has not been the case in HCV-positive recipients for whom outcome has remained unchanged or even worsened over time [14–17].

 This background indicates the importance of identifying the factors related to severe recurrent hepatitis C and monitoring disease progression.

## 3. Factors Associated with the Outcome of HCV-Infected Recipients

In the transplant setting, many factors contribute to disease progression compared with nontransplant patients [10], including, in addition to viral-related factors, donor and recipient-related factors, graft and surgical factors, and immunosuppressive agents (Table 1). Although numerous studies have examined this issue, nearly all have, unfortunately, been retrospective, conducted in limited populations and at single centers, utilized immunosuppressive therapies in an uncontrolled manner, and failed to utilize protocol biopsy to evaluate histologic progression. Yet, investigation of the prognostic factors of severe recurrent disease is important for identifying potential factors for modifying disease outcome and improving organ allocation.

### 3.1. Donor Age

The impact of donor age on outcome has gained increased attention due to the increased use of liver grafts from older donors, which reflects the absolute shortage of available organs. Accumulating data indicates that grafts from older donors are at greater risk for severe histologic findings, disease progression, and impaired graft/patient survival compared with those from younger donors [13, 15, 18–25]. In addition, older donor age might hinder the efficacy of posttransplant antiviral treatment [26–28]. Features of older grafts, such as telomere shortening, impaired hepatocyte proliferation, increased fibrogenesis, and immunologic problems, are thought to be the cause of the lower quality of grafts from aged donors [29]. Recently, Avolio et al. [30] reported that the model for end-stage liver disease (MELD) score adjusted by donor age (D-MELD; calculated as Donor age × MELD) could accurately predict the outcome of HCV-infected recipients.

### 3.2. Graft Characteristics

The use of extended criteria for the donors is especially important for HCV-positive recipients, although studies evaluating long-term outcomes in HCV-positive recipients are lacking. Several studies revealed that grafts from HCV-positive donors could be used as safely as those from HCV-negative donors for hepatitis C cirrhosis [2, 17, 22, 31–34]. Considering superinfection and the impaired response of genotype 1 to antiviral treatment, it is recommended that HCV-positive grafts be used only in HCV genotype 1-positive recipients.

On the other hand, ischemic injury to the graft seems to have a serious impact on patient/graft survival and disease progression [35–39]. An increased risk of severe recurrence of hepatitis C is reported with cardiac death allografts [40], but the most recent analysis of the UNOS/OPTN database revealed the opposite results, and concluded that the use of liver grafts from cardiac death donors is a valuable option for HCV-positive recipients [41].

As for steatosis of graft, despite early studies associating graft steatosis with poor function [42, 43], the impact of allograft steatosis for fibrosis progression and outcome in HCV-positive recipients is unclear [33, 44]. The most recent study by Subramanian et al. [45] indicated that fatty grafts might contribute to fibrosis and poor outcome in HCV-infected recipients. Another recent study by Brandman et al. [23] associated graft steatosis with severe fibrosis at 1 year.

Additionally, several experienced centers reported that LDLT could be performed as safely as deceased donor liver transplantation (DDLT) with an equivalent outcome for HCV-positive recipients [21, 46–50].

### 3.3. Pretransplant Recipient Characteristics

Studies evaluating the association between the severity of HCV recurrence and HCV genotype are conflicting. Some studies suggest that genotype 1b is associated with a poorer outcome [21, 26, 51–53], but other recent studies have not confirmed this finding. Several studies demonstrated that pretransplant HCV RNA in the serum is associated with increased mortality and graft loss [16, 54–56]. It has been also suggested that a less complex quasispecies composition before transplantation is associated with a more severe recurrence [56–58]. Older recipient age [51, 59, 60], race (white donor/black recipient) [61–63], and sex (male [64]/female [15, 65] recipient) are also reported to be associated with impaired outcome. Recent studies suggest that polymorphisms close to the interleukin (IL)-28B gene, both in the recipient and the donor, can affect not only the course of recurrent HCV hepatitis but also the response to antiviral therapy after liver transplantation with a poorer outcome in the CT and TT genotypes than in the CC genotype [66–69], which could be useful for selecting a suitable donor for HCV-infected recipients.

The coexistence of hepatocellular carcinoma is reported to have a negative impact on HCV-positive recipient survival [21, 51, 70–76].

 Coinfection of the human immunodeficiency virus (HIV) in patients with HCV cirrhosis, once considered to be a contraindication for liver transplantation, has now gained wider acceptance for liver transplantation, with the introduction of highly active antiretroviral therapy that increases survival of HIV/HCV coinfected patients and makes end-stage HCV cirrhosis the leading cause of death [77]. Studies suggest that liver transplantation in HIV/HCV coinfected patients is safe and that HIV coinfection does not influence the outcome [78–83]. UNOS no longer considers HIV an absolute contraindication for liver transplantation (http://www.hivtransplant.com/) [84]. 

### 3.4. Posttransplant Recipient Characteristics

Early high viral loads at 7 days [7, 85], 4 months [55, 86], and 12 months posttransplantation [87, 88] are associated with lower patient and graft survival. A recent study by Shackel et al. [88] demonstrated a linear association between viral titers at 12 months and patient survival.

 Postoperative infection with cytomegalovirus (CMV) is associated with more severe HCV disease, increased progression to cirrhosis, and a higher rate of graft failure compared to those without CMV infection [17, 51, 86, 89–92].

Metabolic syndrome occurs in half of HCV-infected recipients within the first 12 months after transplantation and is associated with a greater progression of fibrosis [86]. Several studies demonstrated that posttransplant diabetes in HCV-infected recipients increases the risk of fibrosis/cirrhosis [93–96], but conflicting results have been reported [97]. A causal relationship rather than an association between HCV and diabetes was strongly suggested by a study of 28,942 kidney transplant recipients [98], and accumulating evidence indicates that HCV induces insulin resistance by a variety of mechanisms, which should alert clinicians to the importance of minimizing diabetogenic drugs in the transplant population together with aggressive diabetic control [96]. A recent study by Veldt et al. [99] revealed that increased insulin resistance is associated with a higher rate of advanced fibrosis/cirrhosis in HCV-infected recipients.

## 4. Immunosuppression and Recurrent Hepatitis C

It is generally accepted that over-immunosuppression, such as steroid bolus and OKT3 as rejection therapy, and maintenance immunosuppression with triple-quadruple therapies at full dose are risk factors for HCV liver injury and are associated with a poorer outcome. The optimal immunosuppressive regimen for HCV-infected patients after liver transplantation remains unclear, however, despite several advances in our knowledge regarding the impact of various medications on HCV recurrence in parallel with the development of promising new drugs.

### 4.1. Steroid Boluses and OKT3

Numerous early studies clearly demonstrated that steroid boluses and/or OKT3 administered for graft rejection in HCV-positive patients accelerate recurrent hepatitis C [2, 16, 17, 76, 100–103].

#### 4.1.1. Steroid Maintenance

Based on early perceptions that a steroid bolus for acute rejection accelerates hepatitis C progression, steroids were believed to increase HCV injury. Considering liver injury and the long-term side effects of steroids, steroids were routinely discontinued by 3 months in most liver transplant programs until 2002 [104]. Another option to avoid the negative effects of steroids is to use a steroid-free immunosuppressive regimen.

In addition to early reports [53, 76], two recent retrospective studies [105, 106] revealed that slow steroid tapering (over 6 months) might be associated with less severe recurrent disease. The most compelling data supporting the beneficial effects of low-dose steroids is from Vivarelli et al. [107], who reported the results of a randomized study of rapid (3 months) versus slow (25 months) steroid tapering in conjunction with tacrolimus. The rates of histologic recurrence at the 1-year followup and of advanced fibrosis at the 2-year followup were significantly higher in the rapid tapering group. This important finding might resolve the controversy about the impact of low-dose steroids on the natural history of recurrent hepatitis C.

 Several studies, including a meta-analysis, have demonstrated that steroid-free protocols are not significantly different from other protocols with regard to viremia, patient survival, or fibrosis progression [108–114]. Manousou et al. [115] reported significantly more severe fibrosis in a group receiving tacrolimus monotherapy compared to those receiving triple immunosuppression with azathioprine and short-term steroids, but a recent randomized multicenter study reported that although steroid-free immunosuppression is safe and effective for liver transplant recipients with hepatitis C, steroid-free protocols have no advantage over traditional immunosuppression [116]. Considering the well-known diabetogenic complications of steroids, especially when tacrolimus is the primary immunosuppressive agent, the role of long-term steroid administration remains an important and difficult problem that requires further investigation. Current opinion regarding steroid use in HCV-positive recipients is that steroid boluses should be avoided in cases of mild rejection, steroid-free regimens are safe, and, when steroids are used, withdrawal should be extended with complete discontinuation not before 6 months.

### 4.2. Calcineurin Inhibitors

In vitro series revealed that cyclosporine inhibits HCV replication in a cell-based replicon model [117–119]. Several studies with small populations have confirmed this in vivo series [26, 120–123]. Recently, Spanish groups performing a multicenter retrospective analysis reported that the use of cyclosporine-based immunosuppression regimens and longer treatment duration may protect patients against viral relapse after antiviral treatment [124]. Larger studies reported comparable, even improved results, in a tacrolimus group. Martin et al. [125] found a significantly increased viral load in patients receiving cyclosporine, without any difference in fibrosis or patient/graft survival. In two large prospective studies comparing cyclosporine and tacrolimus, no difference was observed in HCV-positive patients [126, 127]. Berenguer et al. [128] studied the relationship between calcineurin inhibitors and the development of acute hepatitis, fibrosing cholestatic hepatitis, and severe recurrence by protocol biopsies among 136 cyclosporine and 117 tacrolimus patients, which revealed no difference in any of the evaluated variables or in survival. The same authors performed a meta-analysis comprising 366 HCV-positive recipients (183 with tacrolimus, and 183 with cyclosporine) from 5 studies, which revealed no difference in patient or graft survival [129]. The most recent large retrospective study based on the UNOS/OPTN database by Irish et al. [130] analyzed patient death, graft failure, failure due to recurrent disease, and acute cellular rejection among 8092 tacrolimus patients and 717 cyclosporine patients. The findings revealed an increased risk of patient death, graft failure, and acute rejection in the cyclosporine group while the 3-year unadjusted patient and graft survival were comparable, and concluded that the targeted administration of cyclosporine in HCV-infected recipients should be reconsidered. To date, the use of specific calcineurin inhibitors cannot be recommended based on existing data indicating there are no differences in graft/patient survival nor in the progression of recurrent hepatitis C.

### 4.3. Role of Other Immunosuppressive Agents: Antithymocyte Globulin, IL-2 Receptor Antibodies, Mycophenolate, Azathioprine, and Mammalian Target of Rapamycin (mTOR) Inhibitors

Because induction with OKT3 and alemtuzumab is strongly associated with severe recurrent HCV [131, 132], several alternative regimens have been proposed for induction. Among these regimens, the use of rabbit antithymocyte globulin (ATG) as part of a steroid-free protocol gained popularity because an early randomized controlled trial showed a reduced incidence of recurrent HCV in the ATG group compared with a steroid bolus group [109]. Subsequent studies, however, failed to show a positive impact of ATG induction [133, 134], and at present, there are no data that conclusively show that ATG has a positive impact on HCV recurrence compared with steroid induction. Only a few studies have evaluated the impact of the anti-IL-2-receptor monoclonal antibodies baxiliximab and daclizumab for induction in HCV-positive recipients [113, 116, 135–137]. Three prospective studies [116, 136, 137] evaluating induction with IL-2 receptor antibodies failed to show a positive impact on recurrent disease and patient/graft survival. On the other hand, a retrospective study by Nelson et al. [138] reported more severe hepatitis C recurrence in patients with anti-IL-2 receptor antibody induction with mycophenolate mofetil (MMF) when compared to standard therapy based on tacrolimus and steroids. Until adequately powered randomized controlled trials are performed, the use of monoclonal antibodies in HCV-positive liver transplant should be applied with caution and under the rigor of clinical trials.

Azathioprine and MMF are other immunosuppression maintenance drugs associated with disease progression in HCV-infected recipients. An early prospective study showed no effect of MMF in HCV-infected recipients [139]. Recently, however, several studies have reported favorable results for either adding MMF or substituting MMF for azathioprine for graft/patient survival and fibrosis progression [140–145], while other authors found improved or equal effects of azathioprine on disease progression and patient outcome when compared with MMF [64, 115, 146, 147]. A recent review also advocated reappraisal of azathioprine based on several studies that obtained better results with azathioprine [147]. Thus, the overall intensity of immunosuppression rather than the independent action of either drug may have greater impact on HCV recurrence, as shown in recent randomized studies of triple agents [115, 116].

Although mTOR inhibitors have gained widespread use in selected transplant programs as maintenance agents because of their renal-sparing properties, few studies have evaluated the effect of those drugs on the course of recurrent hepatitis C [148–150]. While findings of a few retrospective studies [149, 150] suggested a beneficial effect, there is little evidence to support its widespread use in recurrent HCV patients until results from well-designed, randomized trials are available.

## 5. Posttransplant Followup and Monitoring of HCV Hepatitis Disease Progression

The risk of progression to cirrhosis can be predicted by the biochemical and histologic recurrence pattern. Aminotransferase peak, bilirubin level, and the presence of biochemical cholestasis are associated with a higher rate of progression to graft cirrhosis [151–153]. Histologic findings from liver biopsies performed in the first 12 months after transplantation are useful for predicting the risk of developing cirrhosis, severity of fibrosis, and graft loss [14, 21, 60, 76, 103, 151, 154, 155]. The presence of histologic recurrence, including cholestasis and hepatocellular ballooning, at an early stage is associated with higher rates of progression to cirrhosis [151]. Moderate-to-severe inflammation in liver biopsies performed within the first 12 months is also predictive of progression to cirrhosis and graft loss [21, 60, 103, 154].

 In this background, posttransplant monitoring with reliable methods is crucial for predicting patient/graft outcome, to make an early diagnosis of disease progression, and to start antiviral treatment at the appropriate time. There are two types of prevalent diagnostic methods for monitoring recurrent hepatitis C after liver transplantation; invasive (liver biopsy and measurement of hepatic venous pressure gradient) and noninvasive (elastography, biochemical serum, and fibrogenesis markers, and predictive mathematical models of fibrosis).

Liver biopsy remains the gold standard and the key diagnostic criterion with which other tests are compared in assessing fibrosis. As discussed above, early studies demonstrated the prognostic value of liver biopsy at the time of recurrence and for monitoring disease progression within the first 12 months. With respect to antiviral treatment, a biopsy is essential not only to assess the severity of hepatitis but also to rule out rejection, and initiating treatment in earlier stages of fibrosis results in improved sustained viral response (SVR) rates [26–28]. Consequently, consecutive follow-up protocol biopsies are now widely accepted and recommended by different transplant teams and societies [1, 2, 17, 156, 157]. In contrast, some clinicians object to sequential protocol biopsy given the known limitations of treatment and difficulty in predicting the future of this unpredictable disease [104]. Recently, measuring hepatic venous pressure gradients during transjugular liver biopsies was reported to have a good correlation with fibrosis progression obtained from liver biopsies [158–160]. Hepatic venous pressure gradients greater than 6 mmHg at 12 months are even better for predicting the future development of hepatic decompensation than liver biopsy (sensitivity/specificity; 92%/88% versus 69%/88%) [158].

The estimation of liver stiffness (measured in kilopascals, kPa) with transient elastography (Fibroscan) has been aggressively investigated and is reported to correlate well with the fibrosis progression of HCV-infected grafts after liver transplantation [161–166]. The best cut-off values for detecting patients with graft fibrosis (stage ≥2 for METAVIR or Scheuer scores and ≥3 for Ishak score) vary among studies between 7.9 and 10.1 kPa, with high positive predictive values (65%–85%), negative predictive values (88%–94%), and good discrimination for significant fibrosis (area under the receiver operating characteristics [ROC] curve: 0.81–0.94). For diagnosis of graft cirrhosis, the cut-off values range from 10.5 to 12.5 kPa with 50% to 74% positive predictive values, 99% to 100% negative predictive values, and 0.87 to 0.99 area under the ROC curve [162–166]. Recently, further evaluation by Carrion et al. [167] indicated that repeated measurements of HCV-infected graft stiffness allow for discrimination between slow and rapid fibrosis progression, and that simple scores, including bilirubin and elastography, or donor age and elastography at 6 months, can accurately predict the risk to develop significant fibrosis or portal hypertension in these patients. Elastography using magnetic resonance imaging was also recently reported to be effective [168].

Other noninvasive methods utilizing biochemical markers and predictive mathematical models of fibrosis have also been investigated [161, 169–175]. These include alanine aminotransferase/aspartate aminotransferase ratio index, aspartate aminotransferase/platelets ratio index, Forns index, Fibrotest, hyaluronic acid, procollagen type IV, YKL-40, and mathematical predictive models utilizing some of aforementioned biomarkers with other serum markers [165, 166, 170–175]. The diagnostic accuracies of these studies are reported to have 40% to 75% positive predictive values, 42% to 93% negative predictive values, and 0.56 to 0.82 area under the ROC curve, none of which seems to improve nor surpass the diagnostic efficacy of elastography.

## 6. Antiviral Treatment 

Strategies to improve the outcomes of liver transplantation in HCV-infected recipients include eradication of the HCV virus before transplantation with the use of pretransplant antiviral treatment, eradication of HCV virus early after transplantation preemptively to prevent graft damage, and treatment for established recurrent hepatitis C in the acute, or more commonly, chronic phase. Regardless of the antiviral treatment timing, interferon (INF), especially pegylated-INF (PEG-INF), in conjunction with ribavirin (RBV) are currently accepted as a standard key drugs according to the perspectives obtained in nontransplant populations.

### 6.1. Pretransplantation Antiviral Therapy

 Antiviral treatment before transplantation is aimed at suppressing HCV viremia in liver transplant candidates, which may reduce or eliminate the risk of recurrent infection and disease progression, but this approach is severely limited by poor liver function, a high prevalence of nonresponders, severe cytopenia, and complications, including life-threatening infections [176]. To date, only five studies [177–181] have been published in this phase with differences in the treatment duration (6–14 months versus 2-3 months) and in regimens used (INF only, INF/RBV, or PEG-INF/RBV). Regardless of the approach used, the results are similar, resulting in the prevention of HCV reinfection in about 20% of treated patients with high discontinuation rate and high-dose reduction rate [176]. Based on these five studies, the best candidates for pretransplant antiviral therapy remain Child-Pugh class A whose virologic response rate is high and in whom the risk of side effects is almost identical to controls. Antiviral therapy is contraindicated for Child-Pugh class C patients considering the high risk of severe infections and low SVR rate. In Child-Pugh class B patients, treatment should be discussed on a case-by-case basis considering factors for a potential response. The combination of PEG-INF and RBV at a standard dose in conjunction with growth factors is recommended, and can be discontinued after 1 to 3 months if there is no response.

### 6.2. Posttransplantation Prophylactic and Preemptive Therapy

Viral kinetic studies demonstrated that viremia is minimal in the anheptic phase and immediately after surgery, but the viral load increases as early as the second posttransplant week, reaching its maximal level between the first and third posttransplant month, with even higher levels than those observed at pretransplant period [5]. Therefore, several studies have reported that “prophylactic” or “preemptive” antiviral treatment should be started during this time to suppress viral replication and disease progression, but the results seem less effective [182, 183]. Studies of hepatitis C antibody therapy in the form of hepatitis C immune globulin or monoclonal antibodies against the E2 region motivated by the success of antihepatitis B immune globulins have been disappointing, with only a transient decrease in liver HCV RNA and serum aminotransferase levels [176, 184, 185]. Thus, prophylactic or preemptive antiviral treatment generally means antiviral treatment with INF/PEG-INF and RBV started at early posttransplant period, without requiring evidence of recurrent hepatitis C. The main drawbacks of this therapy are low applicability due to the existence of cytopenia, renal dysfunction, rejection, or extrahepatic complications, high levels of immunosuppression in this time window, and subsequent high frequency of dose reduction and drug discontinuation. In published studies [186–191] of preemptive antiviral therapy, SVR rates are reported to range from 8% to 34% (5% to 43% for genotype 1 and 14% to 100% for genotypes 2 or 3). The rates of dose reduction and drug discontinuation are approximately 70% and 30%, respectively. The most recently published prospective, multicenter, randomized study (PHOENIX study) by Bzowej et al. [192] was designed to compare the efficacy, tolerability, and safety of an escalating dose regimen of PEG-INF alpha 2a/RBV for 48 weeks for preemptive antiviral treatment versus no treatment; 55 received preemptive treatment and 60 patients underwent observation only. The primary endpoint was the proportion of patients with significant histologic recurrence 120 weeks postrandomization. Enrollment into the study ended early because of the slow inclusion of patients, indicating the difficulties of initiating antiviral treatment in the early posttransplant period. The median delay from transplantation to initiation of therapy was 111 and 121 days in the prophylaxis and observation arms, respectively, which was significantly longer than in other preemptive antiviral studies. SVR was achieved in 22% of the prophylaxis patients. The rate of marked HCV recurrence at 120 weeks (62% in prophylaxis patients versus 65% in observation patients), the time until the first recurrence of HCV, histologic recurrence grades, and the progression of fibrosis at 120 weeks, as well as patient/graft survival were similar in both study arms in this intention-to-treat analysis. Dose reduction and discontinuation were required in 70% and 28%, respectively, in the preemptive antiviral treatment group. Based on these results, European and United States transplant societies do not support the routine use of preemptive antiviral therapy.

### 6.3. Antiviral Treatment for Established Recurrent Hepatitis C

The most widely accepted and used strategy is initiating antiviral therapy once recurrent hepatitis C in the graft is established by liver biopsies. Initial studies of monotherapy with IFN-alpha yielded poor results, with SVR rates lower than 5% [193]. With the addition of RBV to IFN-alpha treatment, there is a noticeable improvement in treatment outcomes with an SVR rate of 17% to 30% [194]. More recently, several centers reported that PEG-INF/RBV treatment with an improved SVR rate which has now become an established treatment for recurrent hepatitis in HCV-positive recipients [194–198].

The recent reports of PEG-INF/RBV treatment are summarized in Table 2 [26–28, 199–223]. Most of the data come from uncontrolled studies with different designs regarding time to start treatment, regimen used, and followup, but treatment duration is generally 48 to 52 weeks. Therefore, the results were also very different, with SVR rates ranging 0% to 56% (median: 33%). These results are lower than those obtained in nontransplant populations, possibly due to the immunosuppressive status, high prevalence of genotype 1, high viral load, the difficulty in maintaining adequate antiviral doses (especially RBV), and the difficulty in maintaining therapy for the ideal duration.

Factors affecting SVR rates after PEG-INF/RBV therapy have been aggressively investigated in these studies. Non-1 genotype [26, 27, 199, 202, 213, 218, 220, 234, 235], absence of prior antiviral therapy [194], early virologic response (evaluated after 3 months) [27, 28, 202, 205, 207–210, 214, 215, 217–221, 223, 235], rapid virologic response (evaluated after 1 month) [206, 208, 220], adherence to therapy [27, 202, 207, 211, 213, 216–218], low baseline viral load [27, 208, 211–213, 216, 221, 222], low pretreatment fibrosis stage [26, 28, 204], younger donor age [26, 28, 221, 234], polymorphisms close to the IL-28B gene [66–69], and cyclosporine-based immunosuppression [26, 234, 236] are associated with an improved SVR. Most studies demonstrated improved biochemical and histologic findings, even in virologic nonresponders [222, 237], but whether antiviral therapy slows disease progression in nonresponders has not yet been demonstrated. In addition, several recent retrospective studies with a considerable follow-up period revealed improved patient/graft survival in patients with an SVR [26, 73, 238, 239].

In the absence of controlled studies comparing different treatment regimens, it is not currently possible to determine whether to begin treatment with full or reduced doses and increase as tolerated, or whether individualized treatment is beneficial according to viral response kinetics. Therefore, the rules set out for the nontransplant population should be followed, but adherence to treatment is a major issue for posttransplant recipients. Dose reductions of RBV and/or PEG-INF are necessary in approximately 70% of patients and treatment discontinuation in approximately 30% (Table 2). Dose-dependent hemolytic anemia due to RBV is the major cause of dose reduction and treatment discontinuation in transplant recipients. Several authors have initiated RBV at low doses and then escalated according to tolerance in relation to hemoglobin levels and renal function. To avoid dose reduction, and thus achieve improved SVR, many authors used adjunctive therapy with erythropoietin or granulocyte colony stimulating factor (Table 2). While these drugs improve tolerability to antiviral treatment, there are no data confirming that they result in higher efficacy.

 An increased risk of acute rejection in patients treated with PEG-INF/RBV (5%-6%) compared with those with INF/RBV (1%–3%) was suggested by recent systematic reviews [194–197], although controlled studies did not detect any differences in the rejection rate between treated patients and untreated controls [190, 215, 240]. Whether PEG-INF/RBV therapy increases the risk of rejection remains to be investigated, but acute or chronic rejection seems to be frequently associated with concomitant low or negative serum HCV RNA, leading to an improvement in hepatic function after viral clearance, and resulting in lower serum immunosuppressant levels [241–244]. Thus, close monitoring of calcineurin inhibitor levels is necessary during antiviral treatment. Several authors reported cases with immune-mediated hepatitis observed during or shortly after antiviral treatment (mainly after viral clearance) that responded well to increased immunosuppression [245–247]. In patients under antiviral treatment, particularly in those with undetectable HCV RNA, any flare-up in liver enzymes would suggest rejection or “autoimmune hepatitis” and a liver biopsy should be performed.

Based on the present perspectives, it is compelling to conclude that there is currently no evidence to support the recommendation of antiviral treatment for recurrent graft hepatitis C due to the lack of clinical benefit and frequent adverse effects, as concluded by the recent Cochrane meta-analysis [198]. Recent retrospective cohort studies with a considerable follow-up duration found improved patient/graft survival in patients who obtained an SVR after antiviral treatment [26, 73, 238, 239]. Further randomized clinical trials with adequate trial methodology and adequate follow-up duration are necessary to confirm an actual survival benefit of antiviral treatment. At the same time, direct-acting antivirals such as protease, polymerase, or other nonstructural protein inhibitors should be investigated [248–250].

## 7. Living Donor Liver Transplantation in Patients with HCV Cirrhosis

In areas with low deceased donor organ availability like Japan, the indication of LDLT for HCV cirrhosis is similar to that of DDLT [3], whereas in Western countries, LDLT is conducted in an attempt to alleviate the shortage of donor organs and decrease the mortality among patients awaiting transplants. Early studies raised some concerns, however, regarding the outcomes of LDLT in HCV patients, such as a poorer graft outcome and earlier and more aggressive HCV recurrence after LDLT compared with DDLT [224, 225, 227]. Several theories have been proposed to explain the differences in HCV recurrence between LDLT and DDLT recipients. One possible explanation is that the intense hepatocyte proliferation that occurs in partial liver grafts may lead to increased viral translation and replication [225, 251–253]. Genetic donor-recipient similarity is another proposed mechanism for more severe HCV recurrence [254, 255]. Recent studies however, comparing outcomes of LDLT and DDLT in HCV-infected patients have not only failed to identify LDLT as a risk factor for more intense viral recurrence with impaired outcome, but also revealed improved results in LDLT recipients [21, 46–50, 226, 228–233], which do not support the aforementioned speculations. Alternatively, recent studies favored the theory that outcomes of LDLT for HCV cirrhosis could be better than those of DDLT due to the younger donor age and shorter ischemic time of LDLT grafts. The studies comparing outcomes between LDLT and DDLT in HCV-infected recipients are summarized in Table 3. While several studies demonstrated impaired patient/graft survival and severe histologic findings in LDLT [224, 225, 227], the majority of studies reported equal or even improved outcomes both in patient/graft survival and in fibrosis progression in LDLT [21, 46–50, 226, 228–233]. These data should be interpreted with caution, however, because of the important clinical distinction between LDLT and DDLT. At the time of transplantation, DDLT recipients are far sicker than LDLT recipients as represented by a significantly higher MELD score, donor age is higher, and graft ischemic time is longer, as shown in Table 3. All these factors, as discussed earlier, are considered independent prognostic factors for severe HCV recurrence and impaired patient/graft outcome. Additionally, as Terrault et al. [50] reported, the learning curve for the LDLT procedure may have a considerable impact on the outcome of LDLT for HCV cirrhosis. Jain et al. [233], who recently reported that both patient/graft survival and histologic findings are better in LDLT, found in a sub-analysis of the study that adjusting for MELD score (<25) and donor age (<50) resulted in similar outcomes.

Based on accumulating reports comparing LDLT and DDLT for HCV cirrhosis, hepatitis C recurrence by itself does not seem to explain the differences in patient/graft survival between LDLT and DDLT, and even improved outcomes could be achieved in LDLT due to the better quality of the graft and less sick recipient condition at the time of transplantation. Thus, LDLT could be strongly recommended for HCV-positive patients whenever it is available.

## 8. Retransplantation for Graft Failure Due to Recurrent Hepatitis C

Graft reinfection by HCV is universal with a faster progression to fibrosis and cirrhosis compared with nontransplanted patients, and in those with decompensated graft cirrhosis, retransplantation is the only potentially curative option, although HCV infection has been identified as a risk factor in previous studies [12, 256–263]. Recipient and donor age, bilirubin and creatinine levels, UNOS status, MELD score, time to retransplantation (<1 year), and HCV infection have been identified as independent risk factors in these studies. The International Liver Transplantation Society Expert Panel established that bilirubin ≥ 10 mg/dl, creatinine ≥ 2.0 mg/dl, recipient age < 55, donor age > 40, and early HCV recurrence (cirrhosis within 1 year after transplant) are variables associated with a worse outcome after retransplantation [2].

Due to the lack of a clear consensus with a variety of reported factors, several models based on logistic regression analysis of donor and recipient factors have been developed in the decision-making process for elective retransplantation in HCV-infected patients. These models include the Rosen score [264], the MELD score [12, 262, 265], the Child-Turcotte-Pugh score [258, 262, 266], and the Donor Risk Index [267]. Among these, the Rosen score [264], calculated based on recipient age, bilirubin and creatinine levels, and retransplantation interval, is most widely used and validated. Patients with a Rosen score ≤ 16 had the best 1- and 3-year survival rates (75% and 70%, resp.), while patients with a Rosen score ≥ 20.5 had survival rates of only 42% and 38%, respectively. Two recent studies [263, 266] using the Rosen score as a screening tool revealed similar survival rates in HCV-infected patients and non-HCV-infected patient. Overall, liver retransplantation is not contraindicated in HCV-infected patients, yet in patients with a high risk of death after retransplantation (e.g., ≥20.5 in Rosen score) the use of a new organ seems unreasonable.

## 9. Conclusion

Hepatitis C is here to stay and will remain the most common indication for liver transplantation. Physicians treating HCV-infected candidates and recipients of liver transplantation must be aware of important issues that affect the natural history of recurrent HCV. At present, factors modifiable by clinicians include proper graft allocation, preservation injury, immunosuppression, and antiviral treatment, but many factors among these aspects remain to be determined in future well-designed prospective studies. LDLT can be performed as safely and effectively as DDLT for HCV-infected patients in experienced centers.

## Figures and Tables

**Table 1:** Factors associated with the severity of recurrent hepatitis C after liver transplantation.

Variables	Effect on recurrent hepatitis C
Donor and graft factors	
Age	More severe disease (>40, >50, >65)
Steatosis	Few studies
Prolonged ischemic time	More severe disease
HCV+ graft	No influence
Reduced size versus whole liver (LDLT versus DDLT)	No difference
Pretransplant recipient factors	
Genotype 1b	Controversial
Pre-LT higher viral load	Unclear
Age	Few studies
Race	Few studies
Sex	Few studies
HIV coinfection	No influence
IL-28B gene polymorphism	More severe disease in CT and TT genotype
Posttransplant recipient factors	
Post-LT higher viral load	More severe disease
CMV infection	Unclear More severe disease
Diabetes mellitus (metabolic syndrome)	More severe disease
Immunosuppression	
Steroid bolus	More severe disease
OKT3	More severe disease
Maintenance steroid	Severe disease when rapidly tapered
Steroid free regimen	No influence
Tacrolimus versus Cyclosporine	No difference
Anti-IL-2 receptor antibodies	Controversial
Azathioprine	Controversial
Mycophenolate mofetil	Controversial
mTOR inhibitors	Few studies

Abbreviations: CMV: cytomegalovirus; DDLT: deceased donor liver transplantation; HCV: hepatitis C virus; HIV: human immunodeficiency virus; LDLT: living-donor liver transplantation; LT: liver transplantation; mTOR: mammalian target of rapamycin.

**Table 2:** Studies of antiviral treatment with pegylated interferon alpha and ribavirin for established recurrent hepatitis C after liver transplantation.

Author	Year	Included patients (*n*)	Genotype 1 (%)	FCH	PEG-INF alpha (dose)	RBV dose (mg/day)	Time since LT	Treatment duration (months)	Growth factor	SVR, *n* (%)	Discontinuation *n* (%)	Dose reductions *n* (%)
Rodriguez-Luna et al. [199]	2004	19	63	NA	2b: 0.5–1.5 *μ*g/kg per week (*n* = 19)	400 then escalated to 800–1000	4.2 (1–16.2)	12	Yes	5 (26)	7 (38)	NA
Neff et al. [200]	2004	57	98	NA	2b: 1.5 *μ*g/kg per week (*n* = 57)	400–600	23.5 (1.6–84.7)	12	Yes	8 (14)	18 (32)	INF 38 (67), RBV 22 (39)
Ross et al. [201]	2004	16	69	2	2b: 1.5 *μ*g/kg per week (*n* = 16)	800–1200	9.5	12	Yes	0 (0)	8 (50)	INF 12 (75), RBV 13 (81)
Dumortier et al. [202]	2004	20	80	0	2b: 0.5–1 *μ*g/kg per week (*n* = 20)	400	28 (3–103)	12	No	9 (45)	4 (20)	INF 13 (65), RBV 6 (30)
Babatin et al. [203]	2005	13	46	0	2b: 0.9 *μ*g/kg per week (*n* = 13)	600	24 (6–73)	12	Yes	4 (31)	7 (54)	9 (72)
Toniutto et al. [204]	2005	12	100	0	2b: 0.5 *μ*g/kg per week (*n* = 12)	600–800	14 (0.6–60.8)	12	No	1 (8)	7 (58)	11 (92)
Castells et al. [205]	2005	24	10	0	2b: 1.5 *μ*g/kg per week (*n* = 24)	600	3.8 ± 2.2	12	Yes	8 (35)	3 (13)	INF 6 (25), RBV 14 (58)
Biselli et al. [206]	2006	20	80	0	2b: 1 *μ*g/kg per week (*n* = 20)	600	56.5 (13–157)	12	Yes	9 (45)	1 (5)	RBV 7 (35)
Berenguer et al. [207]	2006	36	89	NA	2b: 1.5 *μ*g/kg per week (*n* = 13), 2a: 180 *μ*g/week (*n* = 23)	600–1200	16.6 (2.7–132.6)	12	Yes	18 (50)	17 (47)	19 (53)
Oton et al. [208]	2006	55	91	0	2b: 1.5 *μ*g/kg per week (*n* = 51), 2a: 180 *μ*g/week (*n* = 4)	800–1200	63.3 ± 45.5	G1/4: 12, G2/3: 6	Yes	24 (44)	16 (29)	INF 17 (31), RBV 17 (31)
Mukherjee and Lyden [209]	2006	32	75	NA	2a: 180 *μ*g/week (*n* = 32)	800 then escalated to 1000–1200	16 (2–70)	G1/4: 12, G2/3: 6	Yes	11 (34)	5 (16)	NA
Mukherjee and Lyden [210]	2006	39	79	NA	2b: 1.5 *μ*g/kg per week (*n* = 39)	800	20 (2–168)	G1/4: 12, G2/3: 6	No	13 (33)	17 (44)	NA
Fernández et al. [211]	2006	47	94	10	2b: 1.5 *μ*g/kg per week (*n* = 47)	600–800	32 ± 25	12	Yes	11 (23)	10 (21)	RBV 15 (32)
Neumann et al. [212]	2006	25	80	0	2b: 1 *μ*g/kg per week (*n* = 25)	600	38 (2–108)	12	Yes	9 (36)	1 (4)	INF 15 (52), RBV 9 (36)
Neumann et al. [212]	2006	61	87	0	2b: 1 *μ*g/kg per week (*n* = 61)	600–800	25 (3–131)	G1/4: 12, G2/3: 6	No	17 (28)	9 (15)	48 (79)
Angelico et al. [214]	2007	42	81	0	2a: 180 *μ*g/week (*n* = 21)	200 then escalated to 1200 until tolerated	48 ± 29	12	No	7 (33)	7 (33)	INF 8 (38), RBV 21 (100)
Carrión et al. [215]	2007	81	93	4	2b: 1.5 *μ*g/kg per week (*n* = 54)	400–1200 adjusted for renal function	14.5 (2–38)	12	Yes	18 (33)	21 (39)	INF 13 (24), RBV 36 (67)
Sharma et al. [216]	2007	35	77	1	2b: 1.5 *μ*g/kg per week, 2a: 180 *μ*g/week, (no of patients not stated)	800	16 (1.5–129)	12	Yes	13 (37)	15 (43)	NA
Zimmermann et al. [217]	2007	26	88	0	2a: 90 *μ*g/week for 4 weeks then escalated to 135–180 *μ*g/week (*n* = 26)	600 then escalated to 800–1200	9.4 ± 3.6	12	Yes	5 (19)	3 (12)	17 (65)
Dinges et al. [218]	2009	19	68	NA	2a: 180 *μ*g/week (*n* = 19)	10 mg/kg/day	23 (6–162)	12	Yes	9 (47)	5 (26)	INF 8 (50), RBV 7 (37)
Lodato et al. [219]	2008	53	100	0	2b: 1.0 *μ*g/kg per week (*n* = 53)	8–10 mg/kg/day	14 (3–151)	12	Yes	14 (26)	24 (45)	INF 3 (6), RBV 21 (40)
Roche et al. [27]	2008	133 (29: INF)	75	NA	2b: 0.6–1.5 *μ*g/kg per week (*n* = 55), 2a: 90–180 *μ*g/week (*n* = 49)	1.8–16.9 mg/kg/day	86 (5–231)	12	Yes	58 (44)	41 (38)	INF 41 (38), RBV 80 (60)
Hanouneh et al. [220]	2008	53	79	0	2b: 1.5 *μ*g/kg per week, 2a: 180 *μ*g/week, (no. of patients not stated)	1000–1200	15 (7–39)	12	Yes	19 (35)	14 (26)	31 (58)
Berenguer et al. [28]	2009	107	86	11	2b: 1.5 *μ*g/kg per week (*n* = 41), 2a: 180 *μ*g/week (*n* = 66)	600–1200	21 (2–133)	12	Yes	39 (37)		INF 37 (35), RBV 43 (40)
Selzner et al. [26]	2009	172 (36: INF)	68	6	2b: 1.5 *μ*g/kg per week, 2a: 180 *μ*g/week, (no. of patients not stated)	800–1000	19 (1–149)	12	Yes	86 (50)	29 (17)	80 (47%)
Schmidt et al. [221]	2010	83	88	NA	2b: 1.0 *μ*g/kg per week (*n* = 30), 2a: 180 *μ*g/week (*n* = 53)	400–1000	41 (0.6–144)	12	Yes	31 (26)	24 (29)	49 (51)
Jain et al. [222]	2010	60	93	NA	2b: 1–1.5 *μ*g/kg per week (*n* = 200), 2a: 180 *μ*g/week (*n* = 40)	800	29 ± 28	12	Yes	21 (35)	24 (40)	INF 21 (35), RBV 16 (27)
Al-Hamoudi et al. [223]	2011	25	0 (All genotype 4)	NA	2a: 180 *μ*g/week (*n* = 25)	400–1200	14 (1–72)	12	Yes	14 (56)	1 (4)	INF 0 (0), RBV 7 (28)

Abbreviations: FCH: fibrosing cholestatic hepatitis; INF: interferon; LT: liver transplantation; NA: not available; PEG-INF: pegylated-interferon; RBV: ribavirin; SVR: sustained viral response.

**Table 3:** Studies comparing living donor liver transplantation and deceased-donor liver transplantation in patients with hepatitis C cirrhosis.

Author	Year	*n* (LDLT/DDLT)	MELD score (LDLT/DDLT)	Donor age (LDLT/DDLT)	Cold ischemia time (h) (LDLT/DDLT)	Follow up (mo)	Histologic progression	Patient survival LDLT/DDLT (%)	Graft survival LDLT/DDLT (%)	Comments
Gaglio et al. [224]	2003	68 (23/45)	12.6/28^∗^	NA	NA	24	NA	87/89	87/85	No difference in outcomes, increased risk of cholestatic hepatitis in LDLT
Shiffman et al. [46]	2004	76 (23/53)	13.5 ± 1.1/16.2 ± 1.0	47.6 ± 2/47.8 ± 0.8	NA	36	No difference	79/82	76/82	No difference in outcomes
Humar et al. [48]	2005	51 (12/39)	17 (14–27)/24 (17–40)^∗^	37.7 ± 9.2/42.8 ± 16.2^¶^	10.2 ± 4.2/<1^†^	28.3	Significantly severe in DDLT	92/90	NA	LDLT may be at a low risk for HCV recurrence
Garcia-Retortillo et al. [225]	2004	117 (22/95)	11 (5–24)/11 (2–28)	31 (19–58)/47 (13–86)^¶^	NA	22	Significantly severe in LDLT	NA	NA	Severe hepatitis C recurrence in LDLT
Maluf et al. [226]	2005	126 (29/97)	13.2 ± 1.1/21 ± 0.8^∗^	NA	0.6 ± 0.2/7.5 ± 2.8^†^	72	NA	67/70	64/69	No difference in survival, more rejection in DDLT and biliary complications in LDLT
Thuluvath and Yoo [227]	2004	619 (207/412)	NA	35.8 ± 0.4/38.9 ± 18.1^¶^	3.9 ± 7.3/8.4 ± 4.5^†^	24	NA	79/81	74/73	Lower graft survival in LDLT
Russo et al. [228]	2004	4234 (279/3955)	NA (TB, PT and Cre were significantly worse in DDLT)	37/40^¶^	8.1/2.6^†^	24	NA	83/81	72/75	No difference in outcomes
Bozorgzadeh et al. [229]	2004	100 (35/65)	14.9 ± 4/15.9 ± 5.3	34.6 ± 9.7/49.2 ± 20.4	NA	39	No difference	89/75	83/64	No difference in outcomes
Van Vlierberghe et al. [230]	2004	43 (17/26)	15 ± 9/15 ± 8	31 ± 8/48 ± 17	3.1 ± 1.3/11.1 ± 2.6^†^	12	No difference	No difference (Presented with only figure)	No difference (Presented with only figure)	No difference in outcomes in short-term
Schiano et al. [231]	2005	26 (11/15)	14 (9–19)/18 (10–31) *P* = 0.05	33 (20–54)/47 (13–73)	0.6 (0.3–1.0)/10 (4.4–20)^†^	24	NA	73/80	73/80	No difference in survival, accelerated viral load increase in LDLT
Guo et al. [49]	2006	67 (15/52)	16.9 ± 6.9/19.0 ± 8.3	NA	NA	24	No difference	93/96	87/94	No difference in outcomes
Terrault et al. [50]	2007	275 (181/94)	14 (6–40)/18 (7–40)^∗^	38 (19–57)/41 (9–72)	0.8 (0.1–8)/6.7 (0.2–10)^†^	36	No difference	74/82	68/80 f	No significant difference in patient/graft survival in experienced LDLT centers
Schmeding et al. [47]	2007	289 (20/269)	NA	38.6 ± 15.2/44.2 ± 12	NA	60	No difference	Better in DDLT (*P* = 0.011)	Better in DDLT (*P* = 0.006)	LDLT does not increase the risk and severity of HCV recurrence, No difference in patient/graft survival when HCC beyond Milan excluded.
Selzner et al. [232]	2008	201 (46/155)	14 (7–39)/17 (6–40)	38 (19–59)/46 (11–79)^¶^	1.5 (0.5–4.9)/7.5 (1.1–16)^†^	60	Significantly severe in DDLT	84/78	76/74	Donor age, rather than transplant approach affects the progression of HCV
Gallegos-Orozco et al. [21]	2009	200 (32/168)	14.6 ± 4.7/25.5 ± 5.9^∗^	35 ± 12/40 ± 16 *P* = 0.05	NA	60	No difference	81/81	NA	LDLT is a good option for HCV cirrhosis
Jain et al. [233]	2011	100 (35/65)	14.5 ± 3.9/16.8 ± 7.3^∗^	34.3 ± 9.3/47.2 ± 19.8^¶^	11 ± 3.1 in DDLT	84	Significantly severe in DDLT at all time points	77/65	71/46	Both patient/graft survival and histologic findings were better in LDLT

^∗^MELD score is significantly higher in DDLT.

^¶^Donor age is significantly higher in DDLT.

^†^Cold ischemia time is significantly longer in DDLT.

Abbreviations: Cre: creatinine; DDLT: deceased donor liver transplantation; LDLT: living donor liver transplantation; MELD: model for end-stage liver disease; NA: not available; PT: prothombin-time; TB: total bilirubin.

## Abbreviations

ALT: Alanine aminotransferase;
AST: Aspartate aminotrasnsferase;
ATG: Antithymocyte globulin;
CMV: Cytomegarovirus;
DDLT: Deceased donor liver transplantation;
FCH: Fibrosing cholestatic hepatitis;
HCV: Hepatitis C virus;
HIV: Human immunodeficiency virus;
IL: Interleukin;
INF: Interferon;
LDLT: Living donor liver transplantation;
MELD: Model for end-stage liver disease;
MMF: Mycophenolate mofetil;
mTOR: Mammalian target of rapamycin;
PEG-INF: Pegylated interferon;
RBV: Ribavirin;
RNA: Ribonucleic acid;
ROC: Receiver operating characteristics;
SVR: Sustained viral response;
UNOS/OPTN: The United Network for Organ Sharing/Organ Procurement and Transplantation Network.

## References

[1] Adam R, McMaster P, O’Grady JG (2003). Evolution of liver transplantation in Europe. Report of the European Liver Transplant Registry. *Liver Transplantation*.

[2] Wiesner RH, Sorrell M, Villamil F (2003). Report of the first international liver transplantation society expert panel consensus conference on liver transplantation and hepatitis C. *Liver Transplantation*.

[3] Sugawara Y, Makuuchi M (2006). Living donor liver transplantation to patients with hepatitis C virus cirrhosis. *World Journal of Gastroenterology*.

[4] Berenguer M, Charco R, Manuel Pascasio J, Ignacio Herrero J (2012). Spanish society of liver transplantation (SETH) consensus recommendations on hepatitis C virus and liver transplantation. *Liver International*.

[5] Garcia-Retortillo M, Forns X, Feliu A (2002). Hepatitis C virus kinetics during and immediately after liver transplantation. *Hepatology*.

[6] Berenguer M (2002). Natural history of recurrent hepatitis C. *Liver Transplantation*.

[7] Gane EJ, Portmann BG, Naoumov NV (1996). Long-term outcome of hepatitis C infection after liver transplantation. *New England Journal of Medicine*.

[8] Berenguer M, Prieto M, Rayón JM (2000). Natural history of clinically compensated hepatitis C virus-related graft cirrhosis after liver transplantation. *Hepatology*.

[9] Narang TK, Ahrens W, Russo MW (2010). Post-liver transplant cholestatic hepatitis C: a systematic review of clinical and pathological findings and application of consensus criteria. *Liver Transplantation*.

[10] Roche B, Samuel D (2007). Risk factors for hepatitis C recurrence after liver transplantation. *Journal of Viral Hepatitis*.

[11] Forman LM, Lewis JD, Berlin JA, Feldman HI, Lucey MR (2002). The association between hepatitis C infection and survival after orthotopic liver transplantation. *Gastroenterology*.

[12] Ghabril M, Dickson R, Wiesner R (2008). Improving outcomes of liver retransplantation: an analysis of trends and the impact of hepatitis C infection. *American Journal of Transplantation*.

[13] Berenguer M, Prieto M, Juan FS (2002). Contribution of donor age to the recent decrease in patient survival among HCV-infected liver transplant recipients. *Hepatology*.

[14] Thuluvath PJ, Krok KL, Segev DL, Yoo HY (2007). Trends in post-liver transplant survival in patients with hepatitis C between 1991 and 2001 in the United States. *Liver Transplantation*.

[15] Belli LS, Burroughs AK, Burra P (2007). Liver transplantation for HCV cirrhosis: improved survival in recent years and increased severity of recurrent disease in female recipients: results of a long term retrospective study. *Liver Transplantation*.

[16] Berenguer M, Ferrell L, Watson J (2000). HCV-related fibrosis progression following liver transplantation: increase in recent years. *Journal of Hepatology*.

[17] Samuel D, Forns X, Berenguer M (2006). Report of the monothematic EASL conference on liver transplantation for viral hepatitis. (Paris, France, January 12–14, 2006). *Journal of Hepatology*.

[18] Maluf DG, Edwards EB, Stravitz RT, Kauftman HM (2009). Impact of the donor risk index on the outcome of hepatitis C virus-positive Liver transplant recipients. *Liver Transplantation*.

[19] Wali M, Harrison RF, Gow PJ, Mutimer D (2002). Advancing donor liver age and rapid fibrosis progression following transplantation for hepatitis C. *Gut*.

[20] Rifai K, Sebagh M, Karam V (2004). Donor age influences 10-year liver graft histology independently of hepatitis C virus infection. *Journal of Hepatology*.

[21] Gallegos-Orozco JF, Yosephy A, Noble B (2009). Natural history of post-liver transplantation hepatitis C: a review of factors that may influence its course. *Liver Transplantation*.

[22] Khapra AP, Agarwal K, Fiel MI (2006). Impact of donor age on survival and fibrosis progression in patients with hepatitis C undergoing liver transplantation using HCV+ allografts. *Liver Transplantation*.

[23] Brandman D, Pingitore A, Lai JC (2011). Hepatic steatosis at 1 year is an additional predictor of subsequent fibrosis severity in liver transplant recipients with recurrent hepatitis C virus. *Liver Transplantation*.

[24] Rayhill SC, Wu YM, Katz DA (2007). Older donor livers show early severe histological activity, fibrosis, and graft failure after liver transplantation for hepatitis C. *Transplantation*.

[25] Machicao VI, Bonatti H, Krishna M (2004). Donor age affects fibrosis progression and graft survival after liver transplantation for hepatitis C. *Transplantation*.

[26] Selzner N, Renner EL, Selzner M (2009). Antiviral treatment of recurrent Hepatitis C after liver transplantation: predictors of response and long-term outcome. *Transplantation*.

[27] Roche B, Sebagh M, Canfora ML (2008). Hepatitis C virus therapy in liver transplant recipients: response predictors, effect on fibrosis progression, and importance of the initial stage of fibrosis. *Liver Transplantation*.

[28] Berenguer M, Aguilera V, Prieto M (2009). Worse recent efficacy of antiviral therapy in liver transplant recipients with recurrent hepatitis C: impact of donor age and baseline cirrhosis. *Liver Transplantation*.

[29] Hoare M, Das T, Alexander G (2010). Ageing, telomeres, senescence, and liver injury. *Journal of Hepatology*.

[30] Avolio AW, Cillo U, Salizzoni M (2011). Balancing donor and recipient risk factors in liver transplantation: the value of D-MELD with particular reference to HCV recipients. *American Journal of Transplantation*.

[31] Northup PG, Argo CK, Nguyen DT (2010). Liver allografts from hepatitis c positive donors can offer good outcomes in hepatitis C positive recipients: a US national transplant registry analysis. *Transplant International*.

[32] Arenas JI, Vargas HE, Rakela J (2003). The use of hepatitis C-infected grafts in liver transplantation. *Liver Transplantation*.

[33] Berenguer M (2008). Risk of extended criteria donors in hepatitis C virus-positive recipients. *Liver Transplantation*.

[34] Ballarin R, Cucchetti A, Spaggiari M (2011). Long-term follow-up and outcome of liver transplantation from anti-hepatitis C virus-positive donors: a European multicentric case-control study. *Transplantation*.

[35] Watt KDS, Lyden ER, Gulizia JM, McCashland TM (2006). Recurrent hepatitis C posttransplant: early preservation injury may predict poor outcome. *Liver Transplantation*.

[36] Baron PW, Sindram D, Higdon D (2000). Prolonged rewarming time during allograft implantation predisposes to recurrent hepatitis C infection after liver transplantation. *Liver Transplantation*.

[37] Feng S, Goodrich NP, Bragg-Gresham JL (2006). Characteristics associated with liver graft failure: the concept of a donor risk index. *American Journal of Transplantation*.

[38] Briceño J, Ciria R, Pleguezuelo M (2007). Contribution of marginal donors to liver transplantation for hepatitis C virus infection. *Transplantation Proceedings*.

[39] Cameron AM, Ghobrial RM, Yersiz H (2006). Optimal utilization of donor grafts with extended criteria: a single-center experience in over 1000 liver transplants. *Annals of Surgery*.

[40] Hernandez-Alejandro R, Croome KP, Quan D (2011). Increased risk of severe recurrence of hepatitis C virus in liver transplant recipients of donation after cardiac death allografts. *Transplantation*.

[41] Uemura T, Ramprasad V, Hollenbeak CS (2012). Liver transplantation for hepatitis C from donation after cardiac death donors: an analysis of OPTN/UNOS data. *American Journal of Transplantation*.

[42] Strasberg SM, Howard TK, Molmenti EP, Hertl M (1994). Selecting the donor liver: risk factors for poor function after orthotopic liver transplantation. *Hepatology*.

[43] Ploeg RJ, D’Alessandro AM, Knechtle SJ (1993). Risk factors for primary dysfunction after liver transplantation—a multivariate analysis. *Transplantation*.

[44] Nocito A, El-Badry AM, Clavien PA (2006). When is steatosis too much for transplantation?. *Journal of Hepatology*.

[45] Subramanian V, Seetharam AB, Vachharajani N (2011). Donor graft steatosis influences immunity to hepatitis C virus and allograft outcome after liver transplantation. *Transplantation*.

[46] Shiffman ML, Stravitz RT, Contos MJ (2004). Histologic recurrence of chronic hepatitis C virus in patients after living donor and deceased donor liver transplantation. *Liver Transplantation*.

[47] Schmeding M, Neumann UP, Puhl G, Bahra M, Neuhaus R, Neuhaus P (2007). Hepatitis C recurrence and fibrosis progression are not increased after living donor liver transplantation: a single-center study of 289 patients. *Liver Transplantation*.

[48] Humar A, Horn K, Kalis A, Glessing B, Payne WD, Lake J (2005). Living donor and split-liver transplants in hepatitis C recipients: does liver regeneration increase the risk for recurrence?. *American Journal of Transplantation*.

[49] Guo L, Orrego M, Rodriguez-Luna H (2006). Living donor liver transplantation for hepatitis C-related cirrhosis: no difference in histological recurrence when compared to deceased donor liver transplantation recipients. *Liver Transplantation*.

[50] Terrault NA, Shiffman ML, Lok ASF (2007). Outcomes in hepatitis C virus-infected recipients of living donor vs. deceased donor liver transplantation. *Liver Transplantation*.

[51] Feray C, Caccamo L, Alexander GJ (1999). European collaborative study on factors influencing outcome after liver transplantation for hepatitis C. European Concerted Action on Viral Hepatitis (EUROHEP) Group. *Gastroenterology*.

[52] Vargas HE, Laskus T, Wang LF (1998). The influence of hepatitis C virus genotypes on the outcome of liver transplantation. *Liver Transplantation and Surgery*.

[53] Berenguer M, Crippin J, Gish R (2003). A model to predict severe HCV-related disease following liver transplantation. *Hepatology*.

[54] Charlton M, Seaberg E, Wiesner R (1998). Predictors of patient and graft survival following liver transplantation for hepatitis C. *Hepatology*.

[55] Sreekumar R, Gonzalez-Koch A, Maor-Kendler Y (2000). Early identification of recipients with progressive histologic recurrence of hepatitis C after liver transplantation. *Hepatology*.

[56] McCaughan GW, Zekry A (2004). Mechanisms of HCV reinfection and allograft damage after liver transplantation. *Journal of Hepatology*.

[57] Doughty AL, Painter DM, McCaughan GW (2000). Post-transplant quasispecies pattern remains stable over time in patients with recurrent cholestatic hepatitis due to hepatitis C virus. *Journal of Hepatology*.

[58] Lyra AC, Fan X, Lang DM (2002). Evolution of hepatitis C viral quasispecies after liver transplantation. *Gastroenterology*.

[59] Selzner M, Kashfi A, Selzner N (2009). Recipient age affects long-term outcome and hepatitis C recurrence in old donor livers following transplantation. *Liver Transplantation*.

[60] Firpi RJ, Abdelmalek MF, Soldevila-Pico C (2004). One-year protocol liver biopsy can stratify fibrosis progression in liver transplant recipients with recurrent hepatitis C infection. *Liver Transplantation*.

[61] Pang PS, Kamal A, Glenn JS (2009). The effect of donor race on the survival of black Americans undergoing liver transplantation for chronic hepatitis C. *Liver Transplantation*.

[62] Saxena V, Lai JC, O'Leary JG (2012). Recipient-donor race mismatch for African American liver transplant patients with chronic hepatitis C. *Liver Transpl*.

[63] Moeller M, Zalawadia A, Alrayes A, Divine G, Brown K, Moonka D (2010). The impact of donor race on recurrent hepatitis C after liver transplantation. *Transplantation Proceedings*.

[64] Walter T, Dumortier J, Guillaud O, Hervieu V, Scoazec JY, Boillot O (2007). Factors influencing the progression of fibrosis in patients with recurrent hepatitis C after liver transplantation under antiviral therapy: a retrospective analysis of 939 liver biopsies in a single center. *Liver Transplantation*.

[65] Lai JC, Verna EC, Brown RS (2011). Hepatitis c virus-infected women have a higher risk of advanced fibrosis and graft loss after liver transplantation than men. *Hepatology*.

[66] Charlton MR, Thompson A, Veldt BJ (2011). Interleukin-28B polymorphisms are associated with histological recurrence and treatment response following liver transplantation in patients with hepatitis C virus infection. *Hepatology*.

[67] Lange CM, Moradpour D, Doehring A (2011). Impact of donor and recipient IL28B rs12979860 genotypes on hepatitis C virus liver graft reinfection. *Journal of Hepatology*.

[68] Fukuhara T, Taketomi A, Motomura T (2010). Variants in IL28B in liver recipients and donors correlate with response to peg-interferon and ribavirin therapy for recurrent hepatitis C. *Gastroenterology*.

[69] Coto-Llerena M, Crespo G, González P (2012). Determination of IL28B polymorphisms in liver biopsies obtained after liver transplantation. *Journal of Hepatology*.

[70] Verna EC, De Martin E, Burra P (2009). The impact of hepatitis C and biliary complications on patient and graft survival following liver transplantation. *American Journal of Transplantation*.

[71] Bozorgzadeh A, Orloff M, Abt P (2007). Survival outcomes in liver transplantation for hepatocellular carcinoma, comparing impact of hepatitis C versus other etiology of cirrhosis. *Liver Transplantation*.

[72] Moya A, Berenguer M, Aguilera V (2002). Hepatocellular carcinoma: can it be considered a controversial indication for liver transplantation in centers with high rates of hepatitis C?. *Liver Transplantation*.

[73] Firpi RJ, Clark V, Soldevila-Pico C (2009). The natural history of hepatitis C cirrhosis after liver transplantation. *Liver Transplantation*.

[74] Böker KHW, Dalley G, Bahr MJ (1997). Long-term outcome of hepatitis C virus infection after liver transplantation. *Hepatology*.

[75] Melum E, Friman S, Bjøro K (2007). Hepatitis C impairs survival following liver transplantation irrespective of concomitant hepatocellular carcinoma. *Journal of Hepatology*.

[76] Samonakis DN, Triantos CK, Thalheimer U (2005). Immunosuppresion and donor age with respect to severity of HCV recurrence after liver transplantation. *Liver Transplantation*.

[77] Sugawara Y, Tamura S, Kokudo N (2011). Liver transplantation in HCV/HIV positive patients. *World Journal of Gastrointestinal Surgery*.

[78] Duclos-Vallee JC, Falissard B, Samuel D (2011). Liver transplant outcomes in HIV-infected patients: a systematic review and meta-analysis with a synthetic cohort. *AIDS*.

[79] Duclos-Vallee JC, Feray C, Sebagh M (2008). Survival and recurrence of hepatitis C after liver transplantation in patients coinfected with human immunodeficiency virus and hepatitis C virus. *Hepatology*.

[80] Moreno A, Cervera C, Fortun J (2012). Epidemiology and outcome of infections in human immunodeficiency virus/hepatitis c virus-coinfected liver transplant recipients: a FIPSE/GESIDA Prospective Cohort Study. *Liver Transplantation*.

[81] De Vera ME, Dvorchik I, Tom K (2006). Survival of liver transplant patients coinfected with HIV and HCV is adversely impacted by recurrent hepatitis C. *American Journal of Transplantation*.

[82] Fung J, Eghtesad B, Patel-Tom K, DeVera M, Chapman H, Ragni M (2004). Liver transplantation in patients with HIV infection. *Liver Transplantation*.

[83] Wojcik K, Vogel M, Voigt E (2007). Antiviral therapy for hepatitis C virus recurrence after liver transplantation in HIV-infected patients: outcome in the Bonn cohort. *AIDS*.

[84] Sherman NMKemmerandKE (2011). Liver transplantation trends in the HIV population. *Digestive Diseases and Sciences*.

[85] Gane EJ, Naoumov NV, Qian KP (1996). A longitudinal analysis of hepatitis C virus replication following liver transplantation. *Gastroenterology*.

[86] Hanouneh IA, Feldstein AE, McCullough AJ (2008). The significance of metabolic syndrome in the setting of recurrent hepatitis C after liver transplantation. *Liver Transplantation*.

[87] Papatheodoridis GV, Barton SGRG, Andrew D (1999). Longitudinal variation in hepatitis C virus (HCV) viraemia and early course of HCV infection after liver transplantation for HCV cirrhosis: the role of different immunosuppressive regimens. *Gut*.

[88] Shackel NA, Jamias J, Rahman W (2009). Early high peak hepatitis C viral load levels independently predict hepatitis C-related liver failure post-liver transplantation. *Liver Transplantation*.

[89] Rosen HR, Chou S, Corless CL (1997). Cytomegalovirus viremia: risk factor for allograft cirrhosis after liver transplantation for hepatitis C. *Transplantation*.

[90] Burak KW, Kremers WK, Batts KP (2002). Impact of cytomegalovirus infection, year of transplantation, and donor age on outcomes after liver transplantation for hepatitis C. *Liver Transplantation*.

[91] Humara A, Kumar D, Raboud J (2002). Interactions between cytomegalovirus, human herpesvirus-6, and the recurrence of hepatitis C after liver transplantation. *American Journal of Transplantation*.

[92] Teixeira R, Pastacaldi S, Davies S (2000). The influence of cytomegalovirus viraemia on the outcome of recurrent hepatitis C after liver transplantation. *Transplantation*.

[93] Baid S, Cosimi AB, Lin Farrell M (2001). Posttransplant diabetes mellitus in liver transplant recipients: risk factors, temporal, relationship with hepatitis C virus allograft hepatitis, and impact on mortality. *Transplantation*.

[94] Foxton MR, Quaglia A, Muiesan R (2006). The impact of diabetes mellitus on fibrosis progression in patients transplanted for hepatitis C. *American Journal of Transplantation*.

[95] AlDosary AA, Ramji AS, Elliott TG (2002). Post-liver transplantation diabetes mellitus: an association with hepatitis C. *Liver Transplantation*.

[96] Gane EJ (2012). Diabetes mellitus following liver transplantation in patients with hepatitis C virus: risks and consequences. *American Journal of Transplantation*.

[97] Tueche SG (2003). Diabetes mellitus after liver transplant new etiologic clues and cornerstones for understanding. *Transplantation Proceedings*.

[98] Abbott KC, Lentine KL, Bucci JR (2004). Impact of diabetes and hepatitis after kidney transplantation on patients who are affected by hepatitis C virus. *Journal of the American Society of Nephrology*.

[99] Veldt BJ, Poterucha JJ, Watt KDS (2009). Insulin resistance, serum adipokines and risk of fibrosis progression in patients transplanted for hepatitis C. *American Journal of Transplantation*.

[100] Lake JR (2003). The role of immunosuppression in recurrence of hepatitis C. *Liver Transplantation*.

[101] Sheiner PA, Schwartz ME, Mor E (1995). Severe or multiple rejection episodes are associated with early recurrence of hepatitis C after orthotopic liver transplantation. *Hepatology*.

[102] Charlton M, Seaberg E (1999). Impact of immunosuppression and acute rejection on recurrence of hepatitis C: results of the national institute of diabetes and digestive and kidney diseases liver transplantation database. *Liver Transplantation and Surgery*.

[103] Neumann UP, Berg T, Bahra M (2004). Long-term outcome of liver transplants for chronic hepatitis C: a 10-year follow-up. *Transplantation*.

[104] Mukherjee S, Sorrell MF (2008). Controversies in liver transplantation for hepatitis C. *Gastroenterology*.

[105] Berenguer M, Aguilera V, Prieto M (2006). Significant improvement in the outcome of HCV-infected transplant recipients by avoiding rapid steroid tapering and potent induction immunosuppression. *Journal of Hepatology*.

[106] Brillanti S, Vivarelli M, De Ruvo N (2002). Slowly tapering off steroids protects the graft against hepatitis C recurrence after liver transplantation. *Liver Transplantation*.

[107] Vivarelli M, Burra P, Barba GL (2007). Influence of steroids on HCV recurrence after liver transplantation: a prospective study. *Journal of Hepatology*.

[108] Sgourakis G, Radtke A, Fouzas I (2009). Corticosteroid-free immunosuppression in liver transplantation: a meta-analysis and meta-regression of outcomes. *Transplant International*.

[109] Eason JD, Nair S, Cohen AJ, Blazek JL, Loss GE (2003). Steroid-free liver transplantation using rabbit antithymocyte globulin and early tacrolimus monotherapy. *Transplantation*.

[110] Margarit C, Bilbao I, Castells L (2005). A prospective randomized trial comparing tacrolimus and steroids with tacrolimus monotherapy in liver transplantation: the impact on recurrence of hepatitis C. *Transplant International*.

[111] Kato T, Gaynor JJ, Yoshida H (2007). Randomized trial of steroid-free induction versus corticosteroid maintenance among orthotopic liver transplant recipients with hepatitis C virus: impact on hepatic fibrosis progression at one year. *Transplantation*.

[112] Lladó L, Fabregat J, Castellote J (2008). Impact of immunosuppression without steroids on rejection and hepatitis C virus evolution after liver transplantation: results of a prospective randomized study. *Liver Transplantation*.

[113] Filipponi F, Callea F, Salizzoni M (2004). Double-blind comparison of hepatitis C histological recurrence rate in HCV+ Liver Transplant Recipients Given Basiliximab+Steroids or Basiliximab+Placebo, in addition to Cyclosporine and Azathioprine. *Transplantation*.

[114] Segev DL, Sozio SM, Shin EJ (2008). Steroid avoidance in liver transplantation: meta-analysis and meta-regression of randomized trials. *Liver Transplantation*.

[115] Manousou P, Samonakis D, Cholongitas E (2009). Outcome of recurrent hepatitis C virus after liver transplantation in a randomized trial of tacrolimus monotherapy versus triple therapy. *Liver Transplantation*.

[116] Klintmalm GB, Davis GL, Teperman L (2011). A randomized, multicenter study comparing steroid-free immunosuppression and standard immunosuppression for liver transplant recipients with chronic hepatitis C. *Liver Transplantation*.

[117] Nakagawa M, Sakamoto N, Enomoto N (2004). Specific inhibition of hepatitis C virus replication by cyclosporin A. *Biochemical and Biophysical Research Communications*.

[118] Henry SD, Metselaar HJ, Lonsdale RCB (2006). Mycophenolic acid inhibits hepatitis C virus replication and acts in synergy with cyclosporin A and interferon-*α*. *Gastroenterology*.

[119] Watashi K, Hijikata M, Hosaka M, Yamaji M, Shimotohno K (2003). Cyclosporin A suppresses replication of hepatitis C virus genome in cultured hepatocytes. *Hepatology*.

[120] Kim RD, Mizuno S, Sorensen JB, Schwartz JJ, Fujita S (2012). Impact of calcineurin inhibitors on hepatitis C recurrence after liver transplantation. *Digestive Diseases and Sciences*.

[121] Firpi RJ, Zhu H, Morelli G (2006). Cyclosporine suppresses hepatitis C virus in vitro and increases the chance of a sustained virological response after liver transplantation. *Liver Transplantation*.

[122] Firpi RJ, Soldevila-Pico C, Morelli GG (2010). The use of cyclosporine for recurrent hepatitis C after liver transplant: a randomized pilot study. *Digestive Diseases and Sciences*.

[123] Van Der Laan LJW, Hudson M, McPherson S (2010). Results of a two-center study comparing hepatic fibrosis progression in HCV-positive liver transplant patients receiving cyclosporine or tacrolimus. *Transplantation Proceedings*.

[124] Fusté LC (2011). Cyclosporine a-based immunosuppression reduces relapse rate after antiviral therapy in transplanted patients with hepatitis C virus infection: a large multicenter cohort study. *Transplantation*.

[125] Martin P, Busuttil RW, Goldstein RM (2004). Impact of tacrolimus versus cyclosporine in hepatitis C virus-infected liver transplant recipients on recurrent hepatitis: a prospective, randomized trial. *Liver Transplantation*.

[126] O’Grady JG, Hardy P, Burroughs AK (2007). Randomized controlled trial of tacrolimus versus microemulsified cyclosporin (TMC) in liver transplantation: poststudy surveillance to 3 years. *American Journal of Transplantation*.

[127] Levy G, Grazi GL, Sanjuan F (2006). 12-month follow-up analysis of a multicenter, randomized, prospective trial in de novo liver transplant recipients (LIS2T) comparing cyclosporine microemulsion (C2 monitoring) and tacrolimus. *Liver Transplantation*.

[128] Berenguer M, Aguilera V, San Juan F (2010). Effect of calcineurin inhibitors in the outcome of liver transplantation in hepatitis C virus-positive recipients. *Transplantation*.

[129] Berenguer M, Royuela A, Zamora J (2007). Immunosuppression with calcineurin inhibitors with respect to the outcome of HCV recurrence after liver transplantation: results of a meta-analysis. *Liver Transplantation*.

[130] Irish WD, Arcona S, Bowers D, Trotter JF (2011). Cyclosporine versus tacrolimus treated liver transplant recipients with chronic hepatitis C: outcomes analysis of the UNOS/OPTN database. *American Journal of Transplantation*.

[131] Rosen HR, Shackleton CR, Higa L (1997). Use of OKT3 is associated with early and severe recurrence of hepatitis C after liver transplantation. *American Journal of Gastroenterology*.

[132] Marcos A, Eghtesad B, Fung JJ (2004). Use of alemtuzumab and tacrolimus monotherapy for cadaveric liver transplantation: with particular reference to hepatitis C virus. *Transplantation*.

[133] Nair S, Loss GE, Cohen AJ, Eason JD (2006). Induction with rabbit antithymocyte globulin versus induction with corticosteroids in liver transplantation: impact on recurrent hepatitis C virus infection. *Transplantation*.

[134] De Ruvo N, Cucchetti A, Lauro A (2005). Preliminary results of a “prope” tolerogenic regimen with thymoglobulin pretreatment and hepatitis C virus recurrence in liver transplantation. *Transplantation*.

[135] Calmus Y, Scheele JR, Gonzalez-Pinto I (2002). Immunoprophylaxis with basiliximab, a chimeric anti-interleukin-2 receptor monoclonal antibody, in combination with azathioprine-containing triple therapy in liver transplant recipients. *Liver Transplantation*.

[136] Klintmalm GBG, Washburn WK, Rudich SM (2007). Corticosteroid-free immunosuppression with daclizumab in HCV+ liver transplant recipients: 1-year interim results of the HCV-3 study. *Liver Transplantation*.

[137] Neuhaus P, Clavien PA, Kittur D (2002). Improved treatment response with basiliximab immunoprophylaxis after liver transplantation: results from a double-blind randomized placebo-controlled trial. *Liver Transplantation*.

[138] Nelson DR, Soldevila-Pico C, Reed A (2001). Anti-interleukin-2 receptor therapy in combination with mycophenolate mofetil is associated with more severe hepatitis C recurrence after liver transplantation. *Liver Transplantation*.

[139] Jain A, Kashyap R, Demetris AJ, Eghstesad B, Pokharna R, Fung JJ (2002). A prospective randomized trial of mycophenolate mofetil in liver transplant recipients with hepatitis C. *Liver Transplantation*.

[140] Wiesner RH, Shorr JS, Steffen BJ, Chu AH, Gordon RD, Lake JR (2005). Mycophenolate mofetil combination therapy improves long-term outcomes after liver transplantation in patients with and without hepatitis C. *Liver Transplantation*.

[141] Sánchez-Bueno F, Ortiz ML, Bermejo J (2006). Prognostic factors for hepatitis C recurrence in patients undergoing orthotopic liver transplantation. *Transplant Immunology*.

[142] Manzia TM, Angelico R, Toti L (2011). Long-term, maintenance MMF monotherapy improves the fibrosis progression in liver transplant recipients with recurrent hepatitis C. *Transplant International*.

[143] Kornberg A, Küpper B, Tannapfel A, Hommann M, Scheele J (2005). Impact of mycophenolate mofetil versus azathioprine on early recurrence of hepatitis C after liver transplantation. *International Immunopharmacology*.

[144] Kornberg A, Küpper B, Wilberg J (2007). Conversion to mycophenolate mofetil for modulating recurrent hepatitis C in liver transplant recipients. *Transplant Infectious Disease*.

[145] Bahra M, Neumann UIFP, Jacob D (2005). MMF and calcineurin taper in recurrent hepatitis C after liver transplantation: impact on histological course. *American Journal of Transplantation*.

[146] Zekry A, Gleeson M, Guney S, McCaughan GW (2004). A prospective cross-over study comparing the effect of mycophenolate versus azathioprine on allograft function and viral load in liver transplant recipients with recurrent chronic HCV infection. *Liver Transplantation*.

[147] Germani G, Pleguezuelo M, Villamil F (2009). Azathioprine in liver transplantation: a reevaluation of its use and a comparison with mycophenolate mofetil. *American Journal of Transplantation*.

[148] Kawahara T, Asthana S, Kneteman NM (2011). m-TOR inhibitors: what role in liver transplantation?. *Journal of Hepatology*.

[149] Asthana S, Toso C, Meeberg G (2011). The impact of sirolimus on hepatitis C recurrence after liver transplantation. *Canadian Journal of Gastroenterology*.

[150] Harper SJF, Gelson W, Harper IG, Alexander GJM, Gibbs P (2011). Switching to sirolimus-based immune suppression after liver transplantation is safe and effective: a single-center experience. *Transplantation*.

[151] Rosen HR, Gretch DR, Oehlke M (1998). Timing and severity of initial hepatitis C recurrence as predictors of long-term liver allograft injury. *Transplantation*.

[152] Prieto M, Berenguer M, Rayón JM (1999). High incidence of allograft cirrhosis in hepatitis C virus genotype 1b infection following transplantation: relationship with rejection episodes. *Hepatology*.

[153] Sánchez-Fueyo A, Restrepo JC, Quintó L (2002). Impact of the recurrence of hepatitis c virus infection after liver transplantation on the long-term viability of the graft. *Transplantation*.

[154] Guido M, Fagiuoli S, Tessari G (2002). Histology predicts cirrhotic evolution of post transplant hepatitis C. *Gut*.

[155] Testa G, Crippin JS, Netto GJ (2000). Liver transplantation for hepatitis C: recurrence and disease progression in 300 patients. *Liver Transplantation*.

[156] Berenguer M, Rayón JM, Prieto M (2001). Are posttransplantation protocol liver biopsies useful in the long term?. *Liver Transplantation*.

[157] Sebagh M, Rifai K, Féray C (2003). All liver recipients benefit from the protocol 10-year liver biopsies. *Hepatology*.

[158] Blasco A, Forns X, Carrión JA (2006). Hepatic venous pressure gradient identifies patients at risk of severe hepatitis C recurrence after liver transplantation. *Hepatology*.

[159] Samonakis DN, Cholongitas E, Thalheimer U (2007). Hepatic venous pressure gradient to assess fibrosis and its progression after liver transplantation for HCV cirrhosis. *Liver Transplantation*.

[160] Kalambokis G, Manousou P, Samonakis D (2009). Clinical outcome of HCV-related graft cirrhosis and prognostic value of hepatic venous pressure gradient. *Transplant International*.

[161] Cholongitas E, Tsochatzis E, Goulis J, Burroughs AK (2010). Noninvasive tests for evaluation of fibrosis in HCV recurrence after liver transplantation: a systematic review. *Transplant International*.

[162] Kamphues C, Lotz K, Röcken C (2010). Chances and limitations of non-invasive tests in the assessment of liver fibrosis in liver transplant patients. *Clinical Transplantation*.

[163] Carrión JA, Navasa M, Bosch J, Bruguera M, Gilabert R, Forns X (2006). Transient elastography for diagnosis of advanced fibrosis and portal hypertension in patients with hepatitis C recurrence after liver transplantation. *Liver Transplantation*.

[164] Rigamonti C, Donato MF, Fraquelli M (2008). Transient elastography predicts fibrosis progression in patients with recurrent hepatitis C after liver transplantation. *Gut*.

[165] Corradi F, Piscaglia F, Flori S (2009). Assessment of liver fibrosis in transplant recipients with recurrent HCV infection: usefulness of transient elastography. *Digestive and Liver Disease*.

[166] Harada N, Soejima Y, Taketomi A (2008). Assessment of graft fibrosis by transient elastography in patients with recurrent hepatitis C after living donor liver transplantation. *Transplantation*.

[167] Carríon JA, Torres F, Crespo G (2010). Liver stiffness identifies two different patterns of fibrosis progression in patients with hepatitis C virus recurrence after liver transplantation. *Hepatology*.

[168] Lee VS, Miller FH, Omary RA (2011). Magnetic resonance elastography and biomarkers to assess fibrosis from recurrent hepatitis c in liver transplant recipients. *Transplantation*.

[169] Cross TJS, Jothimani D, Heneghan MA, Harrison PM (2011). Non-invasive assessment of fibrosis in liver grafts due to hepatitis C virus recurrence. *Clinical Transplantation*.

[170] Cross TJS, Calvaruso V, Foxton MR (2010). A simple, noninvasive test for the diagnosis of liver fibrosis in patients with hepatitis C recurrence after liver transplantation. *Journal of Viral Hepatitis*.

[171] Carrión JA, Fernández-Varo G, Bruguera M (2010). Serum fibrosis markers identify patients with mild and progressive hepatitis C recurrence after liver transplantation. *Gastroenterology*.

[172] Benlloch S, Heredia L, Barquero C (2009). Prospective validation of a noninvasive index for predicting liver fibrosis in hepatitis C virus-infected liver transplant recipients. *Liver Transplantation*.

[173] Benlloch S, Berenguer M, Prieto M, Rayón JM, Aguilera V, Berenguer J (2005). Prediction of fibrosis in HCV-infected liver transplant recipients with a simple noninvasive index. *Liver Transplantation*.

[174] Pungpapong S, Nunes DP, Kirshna M (2008). Serum fibrosis markers can predict rapid fibrosis progression after liver transplantation for hepatitis C. *Liver Transplantation*.

[175] Toniutto P, Fabris C, Bitetto D (2007). Role of AST to platelet ratio index in the detection of liver fibrosis in patients with recurrent hepatitis C after liver transplantation. *Journal of Gastroenterology and Hepatology*.

[176] Roche B, Samuel D (2012). Hepatitis C virus treatment pre- and post-liver transplantation. *Liver International*.

[177] Carrión JA, Martínez-Bauer E, Crespo G (2009). Antiviral therapy increases the risk of bacterial infections in HCV-infected cirrhotic patients awaiting liver transplantation: a retrospective study. *Journal of Hepatology*.

[178] Forns X, García-Retortillo M, Serrano T (2003). Antiviral therapy of patients with decompensated cirrhosis to prevent recurrence of hepatitis C after liver transplantation. *Journal of Hepatology*.

[179] Crippin JS, McCashland T, Terrault N, Sheiner P, Charlton MR (2002). A pilot study of the tolerability and efficacy of antiviral therapy in hepatitis C virus-infected patients awaiting liver transplantation. *Liver Transplantation*.

[180] Everson GT, Trotter J, Forman L (2005). Treatment of advanced hepatitis C with a low accelerating dosage regimen of antiviral therapy. *Hepatology*.

[181] Thomas RM, Brems JJ, Guzman-Hartman G, Yong S, Cavaliere P, Van Thiel DH (2003). Infection with chronic hepatitis C virus and liver transplantation: a role for interferon therapy before transplantation. *Liver Transplantation*.

[182] Terrault NA (2003). Prophylactic and preemptive therapies for hepatitis C virus-infected patients undergoing liver transplantation. *Liver Transplantation*.

[183] Roche B, Samuel D (2011). Is early antiviral therapy for recurrent hepatitis C after liver transplantation superior to later treatment? the answer is no. *Liver Transplantation*.

[184] Davis GL, Nelson DR, Terrault N (2005). A randomized, open-label study to evaluate the safety and pharmacokinetics of human hepatitis C immune globulin (Civacir) in liver transplant recipients. *Liver Transplantation*.

[185] Schiano TD, Charlton M, Younossi Z (2006). Monoclonal antibody HCV-AbXTL68 in patients undergoing liver transplantation for HCV: results of a phase 2 randomized study. *Liver Transplantation*.

[186] Shergill AK, Khalili M, Straley S (2005). Applicability, tolerability and efficacy of preemptive antiviral therapy in hepatitis C-infected patients undergoing liver transplantation. *American Journal of Transplantation*.

[187] Mazzaferro V (2001). Prevention of recurrent hepatitis C after liver transplantation with early interferon and ribavirin treatment. *Transplantation Proceedings*.

[188] Tamura S, Sugawara Y, Yamashiki N, Kaneko J, Kokudo N, Makuuchi M (2010). Pre-emptive antiviral therapy in living donor liver transplantation for hepatitis C: observation based on a single-center experience. *Transplant International*.

[189] Kuo A, Tan V, Lan B (2008). Long-term histological effects of preemptive antiviral therapy in liver transplant recipients with hepatitis C virus infection. *Liver Transplantation*.

[190] Chalasani N, Manzarbeitia C, Ferenci P (2005). Peginterferon Alfa-2a for hepatitis C after liver transplantation: two randomized, controlled trials. *Hepatology*.

[191] Singh N, Gayowski T, Wannstedt CF (1998). Interferon-*α* for prophylaxis of recurrent viral hepatitis C in liver transplant recipients: a prospective, randomized, controlled trial. *Transplantation*.

[192] Bzowej N, Nelson DR, Terrault NA (2011). PHOENIX: a randomized controlled trial of peginterferon alfa-2a plus ribavirin as a prophylactic treatment after liver transplantation for hepatitis C virus. *Liver Transplantation*.

[193] Berenguer M (2005). Treatment of hepatitis C after liver transplantation. *Clinics in Liver Disease*.

[194] Wang CS, Ko HH, Yoshida EM, Marra CA, Richardson K (2006). Interferon-based combination anti-viral therapy for hepatitis C virus after liver transplantation: a review and quantitative analysis. *American Journal of Transplantation*.

[195] Berenguer M (2008). Systematic review of the treatment of established recurrent hepatitis C with pegylated interferon in combination with ribavirin. *Journal of Hepatology*.

[196] Xirouchakis E, Triantos C, Manousou P (2008). Pegylated-interferon and ribavirin in liver transplant candidates and recipients with HCV cirrhosis: systematic review and meta-analysis of prospective controlled studies. *Journal of Viral Hepatitis*.

[197] Guillouche P, Féray C (2011). Systematic review: anti-viral therapy of recurrent hepatitis C after liver transplantation. *Alimentary Pharmacology and Therapeutics*.

[198] Gurusamy KS, Tsochatzis E, Davidson BR, Burroughs AK (2010). Antiviral prophylactic intervention for chronic hepatitis C virus in patients undergoing liver transplantation. *Cochrane Database of Systematic Reviews*.

[199] Rodriguez-Luna H, Khatib A, Sharma P (2004). Treatment of recurrent hepatitis C infection after liver transplantation with combination of pegylated interferon *α*2b and ribavirin: an open-label series. *Transplantation*.

[200] Neff GW, Montalbano M, O’Brien CB (2004). Treatment of established recurrent hepatitis C in liver-transplant recipients with pegylated interferon-alfa-2b and ribavirin therapy. *Transplantation*.

[201] Ross AS, Bhan AK, Pascual M, Thiim M, Cosimi AB, Chung RT (2004). Pegylated interferon *α*-2b plus ribavirin in the treatment of post-liver transplant recurrent hepatitis C. *Clinical Transplantation*.

[202] Dumortier J, Scoazec JY, Chevallier P, Boillot O (2004). Treatment of recurrent hepatitis C after liver transplantation: a pilot study of peginterferon alfa-2b and ribavirin combination. *Journal of Hepatology*.

[203] Babatin M, Schindel L, Burak KW (2005). Pegylated-interferon alpha 2b and ribavirin for recurrent hepatities C after liver liver transplantation: from a Canadian experience to recommendations for therapy. *Canadian Journal of Gastroenterology*.

[204] Toniutto P, Fabris C, Fumo E (2005). Pegylated versus standard interferon-*α* in antiviral regimens for post-transplant recurrent hepatitis C: comparison of tolerability and efficacy. *Journal of Gastroenterology and Hepatology*.

[205] Castells L, Vargas V, Allende H (2005). Combined treatment with pegylated interferon (*α*-2b) and ribavirin in the acute phase of hepatitis C virus recurrence after liver transplantation. *Journal of Hepatology*.

[206] Biselli M, Andreone P, Gramenzi A (2006). Pegylated interferon plus ribavirin for recurrent Hepatitis C infection after liver transplantation in naïve and non-responder patients on a stable immunosuppressive regimen. *Digestive and Liver Disease*.

[207] Berenguer M, Palau A, Fernandez A (2006). Efficacy, predictors of response, and potential risks associated with antiviral therapy in liver transplant recipients with recurrent hepatitis C. *Liver Transplantation*.

[208] Oton E, Barcena R, Moreno-Planas JM (2006). Hepatitis C recurrence after liver transplantation: viral and histologic response to full-dose peg-interferon and ribavirin. *American Journal of Transplantation*.

[209] Mukherjee S, Lyden E (2006). Impact of pegylated interferon *α*-2B and ribavirin on hepatic fibrosis in liver transplant patients with recurrent hepatitis C: an open-label series. *Liver International*.

[210] Mukherjee S, Lyden E (2006). Impact of pegylated interferon alfa-2a and ribavirin on hepatic fibrosis in liver transplant patients with recurrent hepatitis C: an open-label series. *Hepato-Gastroenterology*.

[211] Fernández I, Meneu JC, Colina F (2006). Clinical and histological efficacy of pegylated interferon and ribavirin therapy of recurrent hepatitis C after liver transplantation. *Liver Transplantation*.

[212] Neumann U, Puhl G, Bahra M (2006). Treatment of patients with recurrent hepatitis C after liver transplantation with peginterferon alfa-2B plus ribavirin. *Transplantation*.

[213] Picciotto FP, Tritto G, Lanza AG (2007). Sustained virological response to antiviral therapy reduces mortality in HCV reinfection after liver transplantation. *Journal of Hepatology*.

[214] Angelico M, Petrolati A, Lionetti R (2007). A randomized study on Peg-interferon alfa-2a with or without ribavirin in liver transplant recipients with recurrent hepatitis C. *Journal of Hepatology*.

[215] Carrión JA, Navasa M, García-Retortillo M (2007). Efficacy of antiviral therapy on hepatitis C recurrence after liver transplantation: a randomized controlled study. *Gastroenterology*.

[216] Sharma P, Marrero JA, Fontana RJ (2007). Sustained virologic response to therapy of recurrent hepatitis C after liver transplantation is related to early virologic response and dose adherence. *Liver Transplantation*.

[217] Zimmermann T, Böcher WO, Biesterfeld S (2007). Efficacy of an escalating dose regimen of pegylated interferon *α*-2a plus ribavirin in the early phase of HCV reinfection after liver transplantation. *Transplant International*.

[218] Dinges S, Morard I, Heim M (2009). Pegylated interferon-alpha2a/ribavirin treatment of recurrent hepatitis C after liver transplantation. *Transplant Infectious Disease*.

[219] Lodato F, Berardi S, Gramenzi A (2008). Clinical trial: peg-interferon alfa-2b and ribavirin for the treatment of genotype-1 hepatitis C recurrence after liver transplantation. *Alimentary Pharmacology and Therapeutics*.

[220] Hanouneh IA, Miller C, Aucejo FN, Lopez R, Quinn MK, Zein NN (2008). Recurrent hepatitis C after liver transplantation: ON-treatment prediction of response to peginterferon/ribavirin therapy. *Liver Transplantation*.

[221] Schmidt SC, Bahra M, Bayraktar S (2010). Antiviral treatment of patients with recurrent hepatitis c after liver transplantation with pegylated interferon. *Digestive Diseases and Sciences*.

[222] Jain A, Sharma R, Ryan C (2010). Response to antiviral therapy in liver transplant recipients with recurrent hepatitis C viral infection: a single center experience. *Clinical Transplantation*.

[223] Al-Hamoudi W, Mohamed H, Abaalkhail F (2011). Treatment of genotype 4 hepatitis C recurring after liver transplantation using a combination of pegylated interferon alfa-2a and ribavirin. *Digestive Diseases and Sciences*.

[224] Gaglio PJ, Malireddy S, Levitt BS (2003). Increased risk of cholestatic hepatitis C in recipients of grafts from living versus cadaveric liver donors. *Liver Transplantation*.

[225] Garcia-Retortillo M, Forns X, Llovet JM (2004). Hepatitis C recurrence is more severe after living donor compared to cadaveric liver transplantation. *Hepatology*.

[226] Maluf DG, Stravitz RT, Cotterell AH (2005). Adult living donor versus deceased donor liver transplantation: a 6-year single center experience. *American Journal of Transplantation*.

[227] Thuluvath PJ, Yoo HY (2004). Graft and patient survival after adult live donor liver transplantation compared to a matched cohort who received a deceased donor transplantation. *Liver Transplantation*.

[228] Russo MW, Galanko J, Beavers K, Fried MW, Shrestha R (2004). Patient and graft surivival in hepatitis C recipients after adult living donor liver transplantation in the United States. *Liver Transplantation*.

[229] Bozorgzadeh A, Jain A, Ryan C (2004). Impact of hepatitis C viral infection in primary cadaveric liver allograft versus primary living-donor allograft in 100 consecutive liver transplant recipients receiving tacrolimus. *Transplantation*.

[230] Van Vlierberghe H, Troisi R, Colle I, Ricciardi S, Praet M, De Hemptinne B (2004). Hepatitis C infection-related liver disease: patterns of recurrence and outcome in cadaveric and living-donor liver transplantation in adults. *Transplantation*.

[231] Schiano TD, Gutierrez JA, Walewski JL (2005). Accelerated hepatitis C virus kinetics but similar survival rates in recipients of liver grafts from living versus deceased donors. *Hepatology*.

[232] Selzner N, Girgrah N, Lilly L (2008). The difference in the fibrosis progression of recurrent hepatitis C after live donor liver transplantation versus deceased donor liver transplantation is attributable to the difference in donor age. *Liver Transplantation*.

[233] Jain A, Singhal A, Kashyap R, Safadjou S, Ryan CK, Orloff MS (2011). Comparative analysis of hepatitis C recurrence and fibrosis progression between deceased-donor and living-donor liver transplantation: 8-year longitudinal follow-up. *Transplantation*.

[234] Cescon M, Grazi GL, Cucchetti A (2009). Predictors of sustained virological response after antiviral treatment for hepatitis C recurrence following liver transplantation. *Liver Transplantation*.

[235] Raziorrouh B, Jung MC, Schirren CA (2008). Antiviral therapy for recurrent hepatitis C after liver transplantation: sustained virologic response is related to genotype 2/3 and response at week 12. *European Journal of Gastroenterology and Hepatology*.

[236] Berenguer M, Ortíz-Cantó C, Abellán JJ (2012). Hepatitis C virus viral kinetics during *α*-2a or *α*-2b pegylated interferon plus ribavirin therapy in liver transplant recipients with different immunosuppression regimes. *Journal of Clinical Virology*.

[237] Walter T, Scoazec JY, Guillaud O (2009). Long-term antiviral therapy for recurrent hepatitis C after liver transplantation in nonresponders: biochemical, virological, and histological impact. *Liver Transplantation*.

[238] Veldt BJ, Poterucha JJ, Watt KDS (2008). Impact of pegylated interferon and ribavirin treatment on graft survival in liver transplant patients with recurrent hepatitis C infection. *American Journal of Transplantation*.

[239] Berenguer M, Palau A, Aguilera V, Rayón JM, Juan FS, Prieto M (2008). Clinical benefits of antiviral therapy in patients with recurrent hepatitis C following liver transplantation. *American Journal of Transplantation*.

[240] Samuel D, Bizollon T, Feray C (2003). Interferon-*α* 2b plus ribavirin in patients with chronic hepatitis C after liver transplantation: a randomized study. *Gastroenterology*.

[241] Stravitz RT, Shiffman ML, Sanyal AJ (2004). Effects of interferon treatment on liver histology and allograft rejection in patients with recurrent hepatitis C following liver transplantation. *Liver Transplantation*.

[242] Kugelmas M, Osgood MJ, Trotter JF (2003). Hepatitis C virus therapy, hepatocyte drug metabolism, and risk for acute cellular rejection. *Liver Transplantation*.

[243] Stanca CM, Fiel MI, Kontorinis N, Agarwal K, Emre S, Schiano TD (2007). Chronic ductopenic rejection in patients with recurrent hepatitis C virus treated with pegylated interferon alfa-2a and ribavirin. *Transplantation*.

[244] Selzner N, Guindi M, Renner EL, Berenguer M (2011). Immune-mediated complications of the graft in interferon-treated hepatitis C positive liver transplant recipients. *Journal of Hepatology*.

[245] Cholongitas E, Samonakis D, Patch D (2006). Induction of autoimmune hepatitis by pegylated interferon in a liver transplant patient with recurrent hepatitis C virus. *Transplantation*.

[246] Kontorinis N, Agarwal K, Elhajj N, Fiel MI, Schiano TD (2006). Pegylated interferon-induced immune-mediated hepatitis post-liver transplantation. *Liver Transplantation*.

[247] Berardi S, Lodato F, Gramenzi A (2007). High incidence of allograft dysfunction in liver transplanted patients treated with pegylated-interferon alpha-2b and ribavirin for hepatitis C recurrence: possible de novo autoimmune hepatitis?. *Gut*.

[248] Asselah T, Marcellin P (2011). New direct-acting antivirals’ combination for the treatment of chronic hepatitis C. *Liver International*.

[249] Garg V, van Heeswijk R, Eun Lee J, Alves K, Nadkarni P, Luo X (2011). Effect of telaprevir on the pharmacokinetics of cyclosporine and tacrolimus. *Hepatology*.

[250] Charlton M (2011). Telaprevir, boceprevir, cytochrome P450 and immunosuppressive agents—a potentially lethal cocktail. *Hepatology*.

[251] Fausto N, Campbell JS (2003). The role of hepatocytes and oval cells in liver regeneration and repopulation. *Mechanisms of Development*.

[252] Zimmerman MA, Trotter JF (2003). Living donor liver transplantation in patients with hepatitis C. *Liver Transplantation*.

[253] Olthoff KM (2003). Hepatic regeneration in living donor liver transplantation. *Liver Transplantation*.

[254] Everson GT, Trotter J (2003). Role of adult living donor liver transplantation in patients with hepatitis C. *Liver Transplantation*.

[255] Manez R, Mateo R, Tabasco J, Kusne S, Starzl TE, Duquesnoy RJ (1995). The influence of HLA donor-recipient compatibility on the recurrence of HBV and HCV hepatitis after liver transplantation. *Transplantation*.

[256] Carrión JA, Navasa M, Forns X (2010). Retransplantation in patients with hepatitis C recurrence after liver transplantation. *Journal of Hepatology*.

[257] Yoo HY, Maheshwari A, Thuluvath PJ (2003). Retransplantation of liver: primary graft nonfunction and hepatitis C virus are associated with worse outcome. *Liver Transplantation*.

[258] Neff GW, O’Brien CB, Nery J (2004). Factors that identify survival after liver retransplantation for allograft failure caused by recurrent hepatitis C infection. *Liver Transplantation*.

[259] Pelletier SJ, Schaubel DE, Punch JD, Wolfe RA, Port FK, Merion RM (2005). Hepatitis C is a risk factor for death after liver retransplantation. *Liver Transplantation*.

[260] Roayaie S, Schiano TD, Thung SN (2003). Results of retransplantation for recurrent hepatitis C. *Hepatology*.

[261] Rosen HR, Madden JP, Martin P (1999). A model to predict survival following liver retransplantation. *Hepatology*.

[262] Yao FY, Saab S, Bass NM (2004). Prediction of survival after liver retransplantation for late graft failure based on preoperative prognostic scores. *Hepatology*.

[263] McCashland T, Watt K, Lyden E (2007). Retransplantation for hepatitis C: results of a U.S. multicenter retransplant study. *Liver Transplantation*.

[264] Rosen HR, Prieto M, Casanovas-Taltavull T (2003). Validation and refinement of survival models for liver retransplantation. *Hepatology*.

[265] Watt KDS, Lyden ER, McCashland TM (2003). Poor survival after liver retransplantation: is hepatitis C to blame?. *Liver Transplantation*.

[266] Martí J, Charco R, Ferrer J (2008). Optimization of liver grafts in liver retransplantation: a European single-center experience. *Surgery*.

[267] Northup PG, Pruett TL, Kashmer DM, Argo CK, Berg CL, Schmitt TM (2007). Donor factors predicting recipient survival after liver retransplantation: the retransplant donor risk index. *American Journal of Transplantation*.

